# Evolution of costly signaling and partial cooperation

**DOI:** 10.1038/s41598-019-45272-2

**Published:** 2019-06-19

**Authors:** Mohammad Salahshour

**Affiliations:** Department of Physics, Sharif University of Technology, P.O. Box 11165-9161, Tehran, Iran

**Keywords:** Evolutionary theory, Population dynamics, Computational models, Biological physics, Statistical physics

## Abstract

Two seemingly unrelated, but fundamental challenges in evolutionary theory, are the evolution of costly signals and costly cooperative traits, both expected to reduce an individual’s fitness and diminish by natural selection. Here, by considering a well mixed population of individuals who produce signals and decide on their strategies in a game they play, based on the signals, we show that costly signals and costly cooperative strategies can co-evolve as a result of internal dynamics of the system. Costly signals evolve, despite their apparent cost, due to a favorable cooperative response they elicit. This favorable strategic response can be quantified in a fitness term which governs the distribution of costly signals better than their apparent cost. In the same way, cooperative strategies evolve as they can reach a high fitness due to the internal dynamics of the systems.

## Introduction

Evolution and maintenance of costly signals across many biological organisms have posed a fundamental challenge in evolutionary biology, since the time of Darwin^1^. Costly signaling theory, originated from Zahavi’s handicap principle^1,2^, and later refined by Grafen^3^, aims to explain the evolution of costly signals by linking them to honest communication^1–4^. According to this theory, the function of high cost of signals is to grantee the honesty of signals, as a high production cost makes them expensive to fake^1,2^. Thus, for example, it is argued, wasteful ornaments in many species such as peacock’s tail, which are costly as they are expected to have metabolic cost and increase predation risk, are honest signals which advertise the signal producer’s quality as a potential mate^5^. Similarly, begging calls of birds^6^ or alarm calls when detecting predator^7,8^, both being costly, for example by increasing predation risk, are honest signals of respectively, need and danger. In the same way, it is argued many costly signals observed in humans, such as wasteful signals of wealth (e.g. conspicuous consumption)^9–11^, foraging strategies and generous donation^12–14^, or risky behavior in humans^15,16^, serve a similar function and are honest signals of quality of the producer of the signals.

A seemingly unrelated challenge in evolutionary theory is the evolution of cooperative strategies, despite the cost of cooperation they impose on their bearer^17,18^. As a result of many attempts devoted to explain the evolution of cooperation, some mechanisms are identified which can promote cooperation^17–19^. Kin selection is usually appealed to explain cooperation among close relatives, such as in eusocial insects^20^. Group selection can explain cooperation in relatively closed groups^21,22^. Direct reciprocity^23^ can promote cooperation when interactions are repeated, by retaliation against a selfish act. Indirect reciprocity promotes cooperation through mechanisms such as reputation effects^24,25^. In structured populations, network reciprocity can promote cooperation with certain dynamical rules^26,27^. Tag based mechanisms promote cooperation by channeling the benefit of cooperative acts towards fellow cooperators^28^. Voluntary participation promotes cooperation, in circumstances where individuals can opt out of the game and resort to a safe income^29,30^. Punishment, although is not shown to be able to promote cooperation, is known to increase cooperation level, if cooperation is already evolved by another mechanism^31–34^, or if supplemented by another mechanism to avoid free riding on punishers^35^. Reward is also shown to have positive effects for the evolution of cooperation^36,37^. Similarly, heterogeneity, such as social diversity^38,39^, aging^40^, or knowledge of the past^41^ can improve cooperation level. In addition, the study of the evolution of cooperation on interdependent networks^42–44^, and also multigames^45^, have led to interesting insights into the evolution of cooperation.

The fact that cooperative strategies and costly signals coexist in many contexts, has led to conjectures and arguments that costly signaling can provide another road to the evolution of cooperation^46,47^. This line of thought parallels costly signaling theory, as it is based on the idea that a cooperative act, being costly, can be seen as a costly signal of the cooperator’s quality^13–15,46^. In this manuscript, going beyond the premises of costly signaling theory and trying to find a new road to the evolution of cooperation, we raise the question whether co-evolution of costly signals and cooperation is possible due to a purely dynamical phenomena? By considering a population of signal producing individuals, who play a game and decide on their strategy based on the signals they produce, we note that, signals and strategies form a complex dynamical system, in which both costly signals and costly cooperative strategies can evolve. Costly signals evolve, despite their apparent cost, as they elicit a favorable cooperative strategic response. This strategic response to signals can be quantified in a fitness term which controls the frequency of signals in the population, better than their apparent cost. Similarly, cooperative strategies can evolve, despite the cost of cooperation they impose, due to a large fitness they reach as a result of complex internal dynamics of the system. In the resulting partial cooperative state, agents coordinate in heterogeneous cooperation-defection strategy pairs. That who cooperates and who defects, is determined based on the signals they show by a set of rules. This set of rules emerges from the internal dynamics of the system and can be seen as a set of moral rules which determines legitimate cooperation and defection and supports a partial cooperation state.

## The Model

To see how costly signaling and cooperation can co-evolve, we consider a well mixed population of individuals. At each time step, individuals are paired at random to play a (two person-two strategy) game. Before each game, each individual produces a signal out of *n* possible signals. For this purpose, each individual *α*, has a probability distribution *P*_*α*_(*σ*), for signal production, such that it produces signal *σ* with probability *P*_*α*_(*σ*). An individual’s strategy is determined based on the combination of its own and its opponent’s signal. Thus, we show individual *α*’s strategy by *s*_*α*_(*σ*_*α*_, *σ*_*β*_). Here, *σ*_*α*_ is individual *α*’s signal, and *σ*_*β*_ its opponent’s signal (individual *β*). Each entry of *s*_*α*_(*σ*_*α*_, *σ*_*β*_) can be either *C* (cooperation) or *D* (defection). For example, *s*_*α*_(*σ*_*α*_, *σ*_*β*_) = *C* means individual *α* cooperates if it produces signal *σ*_*α*_ while its opponent produces signal *σ*_*β*_. Signals have costs. We assume signal costs are distributed uniformly at random in the interval [0,*c*_*max*_]. Individuals receive payoff according to the payoff structure of the game, and pay the cost of the signal they have produced. After each round, the population is updated synchronously. That is, individuals reproduce with a probability proportional to their payoff and the new generation replaces the old one, such that the population size *N* remains fixed. The offspring inherit the signal production distribution *P*(*σ*), and the strategy *s*(*σ*, *σ*′) of their parents. However, with probability *ν*_*σ*_ a mutation in *P*(*σ*) occurs, in which case the probability of production of a randomly chosen signal i, is increased. This is done by setting *P*(*σ*) = (1 − *dσ*)*P*(*σ*) + *dσ*[*i*]. Here, [*i*] is a vector whose *i*th element is 1 and its other elements are zero, and *dσ* can be considered as the strength of mutation. In the same way, with probability *ν*_*s*_, a mutation in strategies occur in which case a randomly chosen entry of the strategy matrix is randomly reset to either *C* or *D*.

## Results

**Figure 1:**
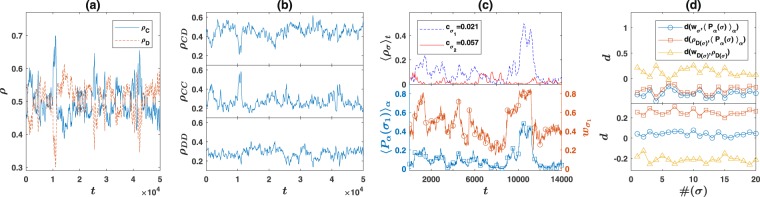
Evolution of partial cooperation and costly signaling. (**a**) Population average of the fraction of cooperation *C* and defection *D* in the strategy matrix of individuals as a function of time. A high level of cooperative strategies is maintained in the population. (**b**) Density of the strategy pairs played in the population, as a function of time. The partial cooperative *CD* strategy pair is the dominant strategy. (**c**) Up: Density of signals produced in the population, for two different signals with shown costs (numbered as signals 1 and 2). Down: Fitness ${w}_{{\sigma }_{1}}$ (red circles), and population average probability of production of signal *σ*_1_, 〈*P*_*α*_(*σ*_1_)〉_*α*_ (blue squares). 〈*P*_*α*_(*σ*)〉_*α*_ follows the fitness of the signal, up to some fluctuations. (**d**) Direction of information flow from *w*_*σ*_ to 〈*P*_*α*_(*σ*)〉_*α*_ (blue circles), from *ρ*_*D*(*σ*)_ to 〈*P*_*α*_(*σ*)〉_*α*_ (red squares), and from the fitness of strategies which defect with signal *σ*, *w*_*D*(*σ*)_ to their density *ρ*_*D*(*σ*)_ (yellow triangles), for different signals numbered from 1 to *n* = 20. In the top panel *ν*_*σ*_ = 0.01, *ν*_*s*_ = 0.05, and in bottom *ν*_*σ*_ = 0.5, *ν*_*s*_ = 0.05. The variable with larger mutation rate becomes slave and the evolution of its density is derived by the other.

We begin by considering a prisoner’s dilemma game (PD). This game is extensively used in studies on the evolution of cooperation^17,19^. See Methods below for payoff and parameter values used in simulations. In Fig. (1a), the fraction of *C* and *D* strategies (the mean fraction of *C* and *D* entries of the strategy matrix *s*_*α*_(*σ*_1_,*σ*_2_) over the whole population), as a function of time is plotted. As can be seen, a high fraction of cooperative strategies, close to $\frac{1}{2}$ is maintained in the population. The fraction of strategy pairs which are actually played, is plotted in Fig. (1b). Both mutual cooperation (*CC*) and mutual defection (*DD*) are maintained and coexist in similar frequencies. However, the strategy pair *CD*, in which an individual cooperates while its opponent defects is the dominant strategy pair most of the times. This means agents somehow coordinate in asymmetric defection and cooperation. To see how this happens, in the top panel of Fig. (1c), we plot the frequency of two of the signals. As can be seen, the signal frequencies show large fluctuations in time. At each instant of time, some of the signals, are produced with smaller frequency compared to others. Consequently, agents lose adaptivity to these signals, and strategies which cooperate with such *rare* signals impose small disadvantage and can increase. When such strategies which cooperate with a rare signal *σ* (we show these strategies by *C*(*σ*)), increase in frequency, signal *σ* reaches a high *fitness* and individuals can achieve high payoff by showing this signal. Consequently, the frequency of signal *σ* increases when *C*(*σ*) strategies are accumulated enough. The individuals who show the signal *σ* can either cooperate or defect. Obviously, those who show signal *σ* and defect achieve a higher payoff compared to those who show signal *σ* and cooperate. This results in higher growth of the former strategies and leads to a high prevalence of the *CD* strategy pair.

This can be seen as a partial solution to the social dilemma. Instead of sticking in the *DD* Nash equilibrium, the system reaches a state in which both *CC* and *CD* strategy pairs are present. This partial solution to cooperation dilemma performs better than the defective *DD* Nash equilibrium. However, still worse than the social optimal *CC* solution. Interestingly however, in a strategic situation where *CD* performs better than *CC*, our model suggests that strategic signaling can provide a way to reach a socially optimal state. Turn Taking Dilemma (TTD) which results from PD by imposing the condition *T* + *S* > 2*R*^48^, is suggested as a game which grasps such strategic situations^48,49^. With this condition, the social optimal is composed of a situation where agents some how manage to coordinate on heterogeneous cooperation-defection. TTD seems to be more difficult than PD to resolve, as it requires agents to solve a coordination task and a cooperation dilemma, at the same time^48^, and no solution to this dilemma in the case of one shot games is known^49,50^. Our study shows signaling can provide a way to naturally resolve such dilemmas in a well mixed population with one shot interactions. TTD is not the only strategic context where heterogeneous *CD* is the socially optimal solution. Prisoner’s dilemma, together with snowdrift (SD), the battle of the sexes (BS), and the leader game, are suggested as the four independent non-trivial archetypal two person, two strategy games^51^. TTD is suggested as a further refinement of this classification^48,49^. Among these, TTD, SD, BS and the leader game, all offer contexts where a heterogeneous *CD* can perform better than (or as good as) a homogeneous strategic pair. In such contexts, individuals need to solve a coordination task to reach a socially optimal state. In TTD and SD, the situation is further complicated as these constitute a social dilemma as well. Signaling, being a natural way to reach a heterogeneous stationary solution, can be appealed to achieve a socially optimal state in all these strategic situations.

As the argument above suggests, even though selection acts on agents, signals and strategies enter a complicated dynamics and can be selected for or against indirectly. To make this statement more quantitative, we define the *fitness* of a signal as the probability that showing that signal results in cooperation by the opponent. This can be calculated as ${w}_{\sigma }={\langle {\sum }_{\sigma ^{\prime} }{P}_{\alpha }(\sigma ^{\prime} ){\delta }_{{s}_{\alpha }(\sigma ^{\prime} ,\sigma ),C}\rangle }_{\alpha }$. Here, 〈.〉_*α*_ denotes an average over population, and ${\delta }_{{s}_{\alpha }(\sigma ^{\prime} ,\sigma ),C}=1$ if *s*_*α*_(*σ*′, *σ*) = *C* and zero otherwise. It is also possible to define a fitness for strategies. Consider the set of strategies which defect against signal *σ*. That is all the strategies of the form *s*(*σ*′, *σ*) = *D*, for all possible *σ*′s (We denote this set by *D*(*σ*)). We define the fitness of such strategies as their expected payoff: ${w}_{D(\sigma )}={\langle {\sum }_{\sigma ^{\prime} }T{P}_{\alpha }(\sigma ){\delta }_{{s}_{\alpha }(\sigma ,\sigma ^{\prime} ),C}{P}_{\beta }(\sigma ^{\prime} )+{\sum }_{\sigma ^{\prime} }P{P}_{\alpha }(\sigma ){\delta }_{{s}_{\alpha }(\sigma ,\sigma ^{\prime} ),D}{P}_{\beta }(\sigma ^{\prime} )\rangle }_{\alpha ,\beta }$. The first term is the probability that an agent shows signal *σ* and cooperates times the temptation *T* (as the payoff of *D*(*σ*) is *T* in this case), and the second term is the probability that an agent shows signal *σ* and defects times punishment *P* (as the payoff of *D*(*σ*) is *P* in this case).

In the lower panel of Fig. (1c), we plot the average probability that a signal (signal *σ*_1_) is produced in the population 〈*P*_*α*_(*σ*_1_)〉_*α*_ (red line marked by circles) together with its fitness (blue line marked with squares), as a function of time. As this example for a signal *σ*_1_ shows, the density of a signal (${\rho }_{{\sigma }_{1}}$ top panel) and 〈*P*_*α*_(*σ*_1_)〉_*α*_ both closely follow its fitness ${w}_{{\sigma }_{1}}$, possibly with a time lag. To look more closely at the co-evolution of signals and strategies, we calculate the direction of information flow from the density of strategies which defect with a signal to the mean probability of production of that signal, denoted as *d*(*ρ*_*D*(*σ*)_, 〈*P*_*α*_(*σ*)〉_*α*_) and plot it in Fig. (1d) (red squares) for different signals numbered from 1 to *n* = 20. *d* always lies between −1 and 1 and a positive *d* means information flows from its first argument (*ρ*_*D*(*σ*)_) to the second (〈*P*_*α*_(*σ*)〉_*α*_), and vice versa [see Methods below]. In the top panel, the mutation rate of signals is smaller than that of strategies (*ν*_*s*_ = 0.05, *ν*_*σ*_ = 0.01), and in the lower panel the mutation rate of signals is larger that that of the strategies (*ν*_*s*_ =  = 0.05, *ν*_*σ*_ = 0.5). As can be seen, whichever has the smaller mutation rate drives the other one, such that the variable with larger mutation rate becomes a fast changing variable and *slave* to the slowly changing variable. In Fig. (1d), the direction of information flow between the fitness and average probability of production of signals *d*(*w*_*σ*_, 〈*P*_*α*_(*σ*)〉_*α*_)) (blue circle), and that between fitness and density of strategies which defect with a given signal *d*(*w*_*D*(*σ*)_, *ρ*_*D*(*σ*)_) (yellow triangles), for different signals are plotted too. We see that the evolution of the slave variable (the one with larger mutation rate) is derived by its fitness (which in turn is determined by the slowly changing variable).

**Figure 2:**
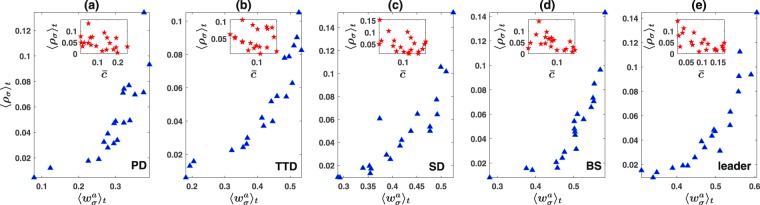
Costly signaling. (**a**) to (**e**) Time average density of signals produced in the population 〈*ρ*_*σ*_〉_*t*_, as a function of their payoff ${\langle {w}_{\sigma }^{a}\rangle }_{t}$ averaged over the same time period for five different game structures. Insets show 〈*ρ*_*σ*_〉_*t*_, as a function of normalized cost $\bar{c}$ (cost divided by mean payoff). When signal densities are plotted against apparent cost, a puzzling picture emerges: not only costly signals are produced, but also signals show little or no dependence on their cost. However, by plotting the signal density against their payoff, a strong pattern emerges: signal densities are distributed as an increasing function of their payoff.

The fact that signals evolve according to their fitness has far reaching consequences; It implies costly signals can evolve, despite having a large apparent cost, as long as they reach a large enough fitness as a result of internal dynamics of the system. Furthermore, this argument implies that signal fitness can explain signal densities better than their apparent cost. This is indeed the case. When the time average density of the signals 〈*ρ*_*σ*_〉_*t*_ is plotted as a function of apparent normalized signal cost $\bar{c}$ (apparent signal cost divided by the mean payoff of the individuals from the games), as in the inset of Fig. (2a) (for different signals numbered from 1 to *n*), a puzzling picture emerges: not only costly signals are produced, despite the fact that they offer no direct benefit and impose a large cost on the signal producer, but also signal density seems almost unrelated to the signal cost (it was the same sort of puzzle which made Darwin to feel sick: “The sight of a feather in a peacock’s tail, whenever I gaze at it, makes me sick” (Letter to Asa Gray, 3 April [1860])). However, when the time average density of the signals is plotted as a function of the time average payoff they accrued (to the signaler) ${\langle {w}_{\sigma }^{a}\rangle }_{t}$, as in Fig. (2a), a strongly increasing function appears, which shows signal densities are determined by their fitness.

This theory is not restricted to the structure of the prisoner’s dilemma game. To see this, we consider all the nontrivial two person two strategy games PD, SD, BS, and the leader game together with the TTD. In Fig. (2b) to Fig. (2e), we perform similar experiments but for respectively, TTD, SD, BS, and the leader game. We see that in all the cases, when the time average frequency of the signals is plotted against apparent cost, a puzzling picture emerges in which signals with large cost are produced. Furthermore, their densities seem to have little or no dependence on their cost (inset of Fig. (2b) to Fig. (2e)). However, when plotted against their time average fitness, an increasing function emerges. We perform a more quantitative test, by examining the existence of a trend between 〈*ρ*_*σ*_〉_*t*_ and their apparent normalized cost $\bar{c}$, and also between 〈*ρ*_*σ*_〉_*t*_ and the time average payoff accrued by signals ${\langle {w}_{\sigma }^{a}\rangle }_{t}$, using both Spearman’s rank correlation and Mann-Kendall tests (see section Methods). The p value of these tests is given in Table (1). As can be seen, while in some cases (TTD and SD) both tests fail to establish a trend between 〈*ρ*_*σ*_〉_*t*_ and $\bar{c}$, in all the cases a strong trend is established between 〈*ρ*_*σ*_〉_*t*_ and ${\langle {w}_{\sigma }^{a}\rangle }_{t}$ (conventionally a trend is considered to be established if the p value of the test is smaller than 0.05). Furthermore, the later trend is significantly stronger than the former in all the cases.

**Table 1:** Trend test between time average density of signals 〈*ρ*_*σ*_〉_*t*_, and normalized apparent cost $\bar{c}$, and between 〈*ρ*_*σ*_〉_*t*_ and time average payoff of signals ${\langle {w}_{\sigma }^{a}\rangle }_{t}$ in different games. From top to down, Spearman’s rank correlation coefficient between 〈*ρ*_*σ*_〉_*t*_ and $\bar{c}$, and between 〈*ρ*_*σ*_〉_*t*_ and ${\langle {w}_{\sigma }^{a}\rangle }_{t}$, p value of the Spearman test between 〈*ρ*_*σ*_〉_*t*_ and $\bar{c}$, and between 〈*ρ*_*σ*_〉_*t*_ and ${\langle {w}_{\sigma }^{a}\rangle }_{t}$, and finally, p value of the Mann-Kendall test between 〈*ρ*_*σ*_〉_*t*_ and $\bar{c}$, and between 〈*ρ*_*σ*_〉_*t*_ and ${\langle {w}_{\sigma }^{a}\rangle }_{t}$. Here, an average over a window of length 5000 time steps is taken. In all the cases both tests strongly support a trend between payoff and density of signals, but in the case of TTD and SD, fail to establish a trend between the apparent cost and density of signals. In all the cases, the trend between density and fitness is significantly stronger compared to the trend between apparent cost and density.

*n*	PD	TTD	SD	BS	leader
$r({\bar{c}}_{\sigma },{\langle {\rho }_{\sigma }\rangle }_{t})$	−0.46	−0.13	−0.37	−0.52	−0.59
$r({\langle {w}_{\sigma }^{a}\rangle }_{t},{\langle {\rho }_{\sigma }\rangle }_{t})$	0.93	0.97	0.92	0.96	0.96
${p}_{r}({\bar{c}}_{\sigma },{\langle {\rho }_{\sigma }\rangle }_{t})$	3.7 × 10^−2^	5.7 × 10^−1^	1.0 × 10^−1^	1.6 × 10^−2^	5.8 × 10^−3^
${p}_{r}({\langle {w}_{\sigma }^{a}\rangle }_{t},{\langle {\rho }_{\sigma }\rangle }_{t})$	2.2 × 10^−9^	6.7 × 10^−13^	5.5 × 10^−9^	8.6 × 10^−12^	3.8 × 10^−12^
${p}_{MK}({\bar{c}}_{\sigma },{\langle {\rho }_{\sigma }\rangle }_{t})$	4.7 × 10^−2^	3.4 × 10^−1^	1.2 × 10^−1^	2.1 × 10^−2^	1.2 × 10^−2^
${p}_{MK}({\langle {w}_{\sigma }^{a}\rangle }_{t},{\langle {\rho }_{\sigma }\rangle }_{t})$	6.9 × 10^−7^	4.1 × 10^−8^	9.6 × 10^−7^	1.7 × 10^−7^	6.0 × 10^−8^

In the Supplementary Information, we develop a mean field theory for a slightly simplified version of the model, which confirms our findings. In addition, we show generality of the results for all parameter values of the model. Furthermore, we investigate the dependence of the level of cooperation on the parameters of the model in both PD and SD games and show a significant level of cooperation is evolved in the population for all parameter values. Finally, we consider a model in which selection occurs with a probability proportional to the exponential of payoff, and show both results, evolution of partial cooperation, and costly signaling hold in that model as well. These establish strategic signaling as a fundamental and novel mechanism for evolution of cooperation, and can explain the presence and maintenance of both costly signals and (costly) cooperative strategies in many biological populations.

## Discussion

The idea that costly signals can help cooperation to flourish had been considered before based on game theoretic arguments^47^. According to this idea, if there are different qualities of individuals to advertise, and if cost and benefit of signals and strategies satisfy certain conditions, in game equilibrium, cooperation can evolve as a costly and honest signal of one’s quality. Such a view is in parallel with costly signaling theory in that it links costly signals to honest communication. In contrast, the mechanism introduced here, shows that costly signals and costly cooperative strategies can co-evolve, in different strategic contexts as modeled by different game structures and under general and broad conditions, due to a purely physical and dynamical phenomena.

The evolution of costly dishonest signals, for example fighting signals and signals of strength^52–55^, or signals of need in sibling conflict^56^, has posed a challenge for costly signaling theory, on the basis of which, some criticism have been raised against costly signaling theory^52^. The dynamical scenario introduced here, instead, shows the evolution of costly signals results from density dependent effects in cost and benefit of signals, and seems to be a simple mechanism which can be argued to be at work in many contexts where costly signals are produced, irrespective of the truthfulness of the signals. This explanation of the evolution of costly signals contrasts costly signaling theory, in that it suggests the evolution of costly signals can have nothing to do with the honesty of communication, and occurs under rather general conditions, as long as individuals decide upon their strategies based on the signals they produce.

One might wonder what advantage costly signals offer? Returning to the top panel of Fig. (1c), we see a key to the answer of this question. Due to having a large cost, individuals tend to produce costly signals less often. Being less frequent, strategies which cooperate with them impose lower fitness cost (compared to the strategies which cooperate with cheep more frequent signals), and thus increase in frequency. This in turn increases the fitness of costly signals in spite of their high cost, and thus increases their frequency, and at the same time increases cooperation level in the system. When increased in frequency, the advantage of costly signals is undermined as the strategies which cooperate with them start to diminish. The competition of agents to maximize their payoff can be considered as a *signaling war*, or an evolutionary arms race between signals and strategies^52^, through which both costly signaling, and cooperation emerges.

## Methods

### Overview of the model

In our model, at each time step agents are randomly paired to play the game. Each pair of individuals *α* and *β*, produce signals, respectively, *σ*_*α*_ and *σ*_*β*_, according to their signal production probabilities and decide about their strategy based on the signals. That is, individual *α* plays strategy *s*_*α*_(*σ*_*α*_, *σ*_*β*_), and individual *β*, plays strategy *s*_*β*_(*σ*_*β*_, *σ*_*α*_). Individuals gather payoff according to the payoff structure of the game and pay the cost of the signals they produce. After playing the game, individuals reproduce according to their net payoff, such that the population size *N* remains constant. In other words, each individual in the next generation is the offspring of an individual *α* in the past generation with probability $\frac{{w}_{\alpha }}{{\sum }_{\alpha \mathrm{=1}}^{N}{w}_{\alpha }}$. Here, *w*_*α*_ is the net payoff of individual *α* (In case an agent’s net payoff becomes negative, it is set to zero. This makes sure that the corresponding agent does not contribute any offspring to the next generation). The offspring inherit the signal production probability *P*(*σ*) and the strategy matrix *s*(*σ*_1_, *σ*_2_) of their parent. However, mutations can occur. With probability *ν*_*σ*_ a mutation in signal production probability occurs in which case the probability that the offspring produces a randomly chosen signal *i* increases. This is done by setting *P*_*o*_(*σ*) = (1 − *dσ*)*P*_*p*_(*σ*) + *dσ*[*i*]. Here, [*i*] is a vector whose *i*th element is 1 and its other elements are zero, and the subindices *o* and *p* refer respectively, to offspring and parent. With probability *ν*_*s*_ a mutation in strategy occurs in which case a randomly chosen entry of the strategy matrix of the offspring is set randomly equal to either *C* or *D*.

### Simulations

The simulations start with random assignment of strategies to individuals. That is, each entry of the strategy matrix of each individual is randomly set equal to either *C* or *D*. The initial signal production probability distribution of each agent *α*, *P*_*α*_(*σ*) is chosen independently of others, and uniformly at random such that the normalization condition holds. That is, ${P}_{\alpha }(\sigma )=\frac{{f}_{\alpha }(\sigma )}{{\sum }_{\sigma }{f}_{\alpha }(\sigma )}$, where *f*_*α*_(*σ*) for (*α* = 1..*N* and *σ* = *σ*_1_..*σ*_*n*_) are random numbers, chosen uniformly at random in the interval [0, 1]. The base parameter values used in the simulations (unless otherwise specified) are as follows: *ν*_*s*_ = 0.05, *ν*_*σ*_ = 0.05, *dσ* = 0.1, *N* = 400, *c*_*max*_ = 0.1. Games and their payoffs are given in Table (2). Direction of information flow plotted in Fig. (1d) is defined as $d(x,y)=\frac{{\sum }_{\tau \mathrm{=1}}^{20}{d}_{5\tau }(x,y)}{20}$, where ${d}_{\tau }(x,y)=\frac{I({x}_{t},{y}_{t+\tau }|{y}_{t})-I({y}_{t},{x}_{t+\tau }|{x}_{t})}{I({x}_{t},{y}_{t+\tau }|{y}_{t})+I({y}_{t},{x}_{t+\tau }|{x}_{t})}$, (*x*_*t*_, etc., are time series and *I* stands for the conditional mutual information)^57^. Time series used are run for 50000 time steps. Time averages in Fig. (2) are taken over a time window of length 5000 time steps starting from *t* = 10000 in the simulations. Trend tests used in Table (1) are performed on the same data.

**Table 2:** Games and their payoffs. R is the payoff to mutual cooperation, T payoff to defection against a cooperator, S payoff to cooperation with a defector, and P the payoff of mutual defection.

	R	S	T	P		R	S	T	P		R	S	T	P
PD	0.6	0	0.8	0.2	TTD	0.6	0	1.4	0.2	SD	0.6	0.4	0.8	0.2
BS	0.4	0.8	0.6	0.2	leader	0.4	0.6	0.8	0.2					

### Trend tests

The Mann-Kendall test is computed as follows. To examine whether time average signal densities 〈*ρ*_*σ*_〉_*t*_ is an increasing (or decreasing) function of another variable, for example normalized signal costs ${\bar{c}}_{\sigma }$, we first order signal densities according to their cost starting from the signal with lowest cost. Denoting the ordered signal densities by *y*_*i*_ for *i* = 1..*n*, the Mann-Kendall statistics is defined as $S={\sum }_{i\mathrm{=1}}^{n}\,{\sum }_{j=i+1}^{n}sgn({y}_{j}-{y}_{i})$, where *sgn*(*x*) = 1 if *x* > 0, *sgn*(*x*) = −1 if *x* < 0, and *sgn*(*x*) = 0 if *x* = 0. The p value of the test is defined as the probability that a value as extreme as *S* is reached under the null hypothesis that no trend exists. For large samples ($n\ge 10$), the Mann-Kendall standardized *Z* statistics, as a normal approximation to *S* is computed as follows, ${Z}_{MK}=\frac{S-1}{{\sigma }_{S}}$ if *S* > 0, ${Z}_{MK}=\frac{S+1}{{\sigma }_{S}}$ if *S* < 0, and *Z*_*MK*_ = 0 if *S* = 0. Where, the standard deviation of *S* is ${\sigma }_{S}=\sqrt{\frac{1}{18}n(n-\mathrm{1)(2}n+\mathrm{5)}-{\sum }_{p\mathrm{=1}}^{g}{t}_{p}({t}_{p}-\mathrm{1)(2}{t}_{p}+\mathrm{5)}}$. Here, *n* is the size of the sample (i.e. number of signals), *g* is the number of tied groups in the sample, and *t*_*p*_ is the size of the *p*th tied group (a tied group is a set of equal data points in the sample, and the size of a tied group is the number of data points belonging to that group). The p value of the test can be calculated using *Z*_*MK*_, as the probability that a value as high as *Z*_*MK*_ is reached under the null hypothesis that no trend exists. This can be determined by referring to statistical tables in statistic textbooks or softwares. See, for example^58^, for further details.

The Spearman rank correlation coefficient between two variables *x* and *y*, *r*(*x*, *y*), is defined as the Pearson’s correlation between rank ordered variables. That is, to calculate the Spearman rank correlation coefficient between *x* and *y*, first their values are converted to their ranks, resulting in the rank ordered variable *Rx* and *Ry*, and then the Pearson’s correlation is calculated as the covariance of the rank variables divided by their standard deviations, $r(x,y)=\frac{cov(Rx,Ry)}{{\sigma }_{Rx}{\sigma }_{Ry}}$. When the ranks of all the variables are distinct (which holds in our case as the variables are real numbers and the probability of ties is almost zero), this can be calculated using the simple formula $r=1-\frac{6{\sum }_{i\mathrm{=1}}^{n}{d}_{i}}{n({n}^{2}-\mathrm{1)}}$. Where, *d*_*i*_ is the difference between ranks of *x*_*i*_ and *y*_*i*_. The p value of the test, defined as the probability that a value as high as *r* is observed under the null hypothesis that no trend exists, is calculated using the variable $t=r\sqrt{\frac{n-2}{1-{r}^{2}}}$, by taking the fact into account that *t* is distributed approximately according to student’s t distribution. This can be determined by referring to statistical tables in many statistic textbooks or software packages. See for example^59^ for further details.

## Supplementary information

Supplementary Information pdf text

## References

[1] Zahavi, A & Zahavi, A. *The Handicap Principle: A Missing Piece Of Darwin’s Puzzle*. (Oxford University Press, 1999).

[2] Zahavi A (1975). Mate selection—a selection for a handicap. Journal of theoretical Biology..

[3] Grafen A (1990). Biological signals as handicaps. Journal of theoretical biology..

[4] Számadó S (2011). The cost of honesty and the fallacy of the handicap principle. Animal Behaviour..

[5] Smith, J. M. & Harper, D. *Animal Signals*. (Oxford University Press, 2003).

[6] Leech SM, Leonard ML (1997). Begging and the risk of predation in nestling birds. Behavioral Ecology..

[7] Smith JM (1965). The evolution of alarm calls. The American Naturalist..

[8] Bergstrom CT, Lachmann M (2001). Alarm calls as costly signals of antipredator vigilance: the watchful babbler game. Animal behaviour..

[9] Sundie JM (2011). Peacocks, Porsches, and Thorstein Veblen: Conspicuous consumption as a sexual signaling system. Journal of personality and social psychology..

[10] Hawkes K, Bird RB (2002). Showing off, handicap signaling, and the evolution of men’s work. Evolutionary Anthropology: Issues, News, and Reviews: Issues, News, and Reviews.

[11] Van Vugt M, Hardy CL (2010). Cooperation for reputation: Wasteful contributions as costly signals in public goods. Group Processes & Intergroup Relations..

[12] Smith EA, Bird RLB (2000). Turtle hunting and tombstone opening: Public generosity as costly signaling. Evolution and Human Behavior..

[13] Bird RB, Smith E, Bird. DW (2001). The hunting handicap: costly signaling in human foraging strategies. Behavioral Ecology and Sociobiology..

[14] Marlowe, F. *The Hadza: Hunter-Gatherers of Tanzania*. Vol. 3. (Univ of California Press, 2010).

[15] Lyle HF, Smith EA, Sullivan RJ (2009). Blood donations as costly signals of donor quality. Journal of Evolutionary Psychology..

[16] Densley JA (2012). Street gang recruitment: Signaling, screening, and selection. Social problems..

[17] Axelrod R, Hamilton WD (1981). The evolution of cooperation. science.

[18] Doebeli M, Hauert C (2005). Models of cooperation based on the Prisoner’s Dilemma and the Snowdrift game. Ecology letters..

[19] Nowak MA (2006). Five rules for the evolution of cooperation. science..

[20] Hamilton WD (1964). The genetical evolution of social behaviour II. Journal of theoretical biology..

[21] Wilson DS (1975). A theory of group selection. Proceedings of the national academy of sciences.

[22] Traulsen A, Nowak MA (2006). Evolution of cooperation by multilevel selection. Proceedings of the National Academy of Sciences..

[23] Trivers RL (1971). The evolution of reciprocal altruism. The Quarterly review of biology..

[24] Nowak MA, Sigmund K (1998). Evolution of indirect reciprocity by image scoring. Nature..

[25] Nowak MA, Sigmund K (2005). Evolution of indirect reciprocity. Nature..

[26] Ohtsuki H, Hauert C, Lieberman E, Nowak MA (2006). A simple rule for the evolution of cooperation on graphs and social networks. Nature..

[27] Szabó G, Fath G (2007). Evolutionary games on graphs. Physics reports..

[28] Riolo RL, Cohen MD, Axelrod R (2001). Evolution of cooperation without reciprocity. Nature..

[29] Hauert C, De Monte S, Hofbauer J, Sigmund K (2002). Volunteering as red queen mechanism for cooperation in public goods games. Science..

[30] Szabó G, Hauert C (2002). Phase transitions and volunteering in spatial public goods games. Physical review letters..

[31] Perc M (2017). Statistical physics of human cooperation. Physics Reports..

[32] Boyd R, Gintis H, Bowles S (2010). Coordinated punishment of defectors sustains cooperation and can proliferate when rare. Science..

[33] Szolnoki A, Perc M (2017). Second-order free-riding on antisocial punishment restores the effectiveness of prosocial punishment. Physical Review X.

[34] Chen X, Szolnoki A, Perc M (2015). Competition and cooperation among different punishing strategies in the spatial public goods game. Physical Review E..

[35] Chen X, Szolnoki A, Perc M (2014). Probabilistic sharing solves the problem of costly punishment. New Journal of Physics.

[36] Szolnoki A, Perc M (2015). Antisocial pool rewarding does not deter public cooperation. Proceedings of the Royal Society B: Biological Sciences..

[37] Hilbe C, Sigmund K (2010). Incentives and opportunism: from the carrot to the stick. Proceedings of the Royal Society B: Biological Sciences..

[38] Santos FC, Santos MD, Pacheco JM (2008). Social diversity promotes the emergence of cooperation in public goods games. Nature..

[39] Perc. M, Szolnoki A (2008). Social diversity and promotion of cooperation in the spatial prisonerâ€™s dilemma game. Physical Review E.

[40] Szolnoki A, Perc M, Szabó G, Stark HU (2009). Impact of aging on the evolution of cooperation in the spatial prisoner’s dilemma game. Physical Review E..

[41] Danku Z, Perc M, Szolnoki A (2019). Knowing the past improves cooperation in the future. Scientific reports..

[42] Szolnoki A, Perc M (2013). Information sharing promotes prosocial behaviour. New Journal of Physics..

[43] Xia C, Li X, Wang Z, Perc M (2018). Doubly effects of information sharing on interdependent network reciprocity. New Journal of Physics..

[44] Shi *et al*. Winner-weaken-loser-strengthen rule leads to optimally cooperative interdependent networks. *Nonlinear Dynamics*. 1–8 (2019).

[45] Li Z (2019). The effect of multigame on cooperation in spatial network. Applied Mathematics and Computation.

[46] Roberts G (1998). Competitive altruism: from reciprocity to the handicap principle. Proceedings of the Royal Society of London. Series B: Biological Sciences..

[47] Gintis H, Smith EA, Bowles S (2001). Costly signaling and cooperation. Journal of theoretical biology..

[48] Neill, D. B. Cooperation and coordination in the turn-taking dilemma. *Proceedings of the 9th Conference on Theoretical Aspects of Rationality and Knowledge*. (pp. 231–244). (ACM, 2003).

[49] Stark HU (2010). Dilemmas of partial cooperation. *Evolution: International*. Journal of Organic Evolution..

[50] Browning L, Colman AM (2004). Evolution of coordinated alternating reciprocity in repeated dyadic games. Journal of Theoretical Biology..

[51] Rapoport A (1967). Exploiter, leader, hero, and martyr: The four archetypes of the 2 × 2 game. Behavioral science..

[52] Dawkins R, Krebs JR (1979). Arms races between and within species. Proceedings of the Royal Society of London. Series B. Biological Sciences..

[53] Lailvaux SP, Reaney LT, Backwell PR (2009). Dishonest signalling of fighting ability and multiple performance traits in the fiddler crab Uca mjoebergi. Functional Ecology..

[54] Backwell PR, Christy JH, Telford SR, Jennions MD, Passmore J (2000). Dishonest signalling in a fiddler crab. Proceedings of the Royal Society of London. Series B: Biological Sciences..

[55] Wilson RS, Angilletta MJ, James RS, Navas C, Seebacher F (2007). Dishonest signals of strength in male slender crayfish (Cherax dispar) during agonistic encounters. The American Naturalist..

[56] Caro SM, West SA, Griffin AS (2016). Sibling conflict and dishonest signaling in birds. Proceedings of the National Academy of Sciences..

[57] Hlaváčkováá-Schindler K, Paluš M, Vejmelka M, Bhattacharya J (2007). Causality detection based on information-theoretic approaches in time series analysis. Physics Reports..

[58] Gilbert, R. O. *Statistical Methods For Environmental Pollution Monitoring*. (John Wiley & Sons, 1987).

[59] Well, A. D. & Myers, J. L. *Research Design & Statistical Analysis*. (Psychology Press, 2003).

