# Supplementary material for: Evolution of costly signaling and partial cooperation
Source: Sci Rep. 2019 Jun 19;9:8792. doi: 10.1038/s41598-019-45272-2 (PMC6584789; doi:10.1038/s41598-019-45272-2)
Supplement: Supplementary file 1 — Supplementary Information pdf text [file 41598_2019_45272_MOESM1_ESM.pdf]

Supplementary Information
Evolution of costly signaling and partial cooperation

Mohammad Salahshour
Department of Physics, Sharif University of Technology, P.O.Box 11155-9161, Tehran, Iran

SI. 1 The Model

We consider a well mixed population of agents. At each time step, agents are randomly paired to play a two person-two strategy game. Each agent α has a probability distribution $P_\alpha(\sigma)$ for signal production, such that it produces signal σ with probability $P_\alpha(\sigma)$. Agents decide on their strategy based on the combination of signals which they and their opponent have produced. In other words, the strategy of an agent α is a function of its own, and its opponent's strategy and is shown by $s_\alpha(\sigma_\alpha, \sigma_\beta)$. Here, σ_α is agent α's strategy, and σ_β is its opponent's (agent β) strategy. Each entry of the strategy matrix can be either C (cooperation) or D (defection). For example $s_\alpha(\sigma_1, \sigma_2) = C$ means agent α cooperates if it produces signal σ_1 and its opponent produces signal σ_2.

At each time step agents are randomly paired to play the game. They gather payoff according to the payoff structure of the game, pay the cost of the signals they produce, and reproduce according to their net payoff. Reproduction step is done in the following manner. After gathering payoff, the whole population is updated. Individuals produce offspring with a probability proportional to their payoff, such that the population size N remains constant. In other words, each individual in the next generation is the offspring of an individual α in the past generation with probability $\frac{w_\alpha}{\sum_{\alpha=1}^{N} w_\alpha}$. Here, w_α is the net payoff of individual α (In case an agent's net payoff becomes negative, it is set to zero. This makes sure that the corresponding agent does not contribute any offspring to the next generation). The offspring inherit the signal production probability $P(\sigma)$ and the strategy matrix $s(\sigma_1, \sigma_2)$ of their parent. However, mutations can occur. With probability ν_σ a mutation in signal production probability occurs in which case the probability that the offspring produces a randomly chosen signal i increases. This is done by setting $P_o(\sigma) = (1 - d\sigma)P_p(\sigma) + d\sigma[i]$. Here, $[i]$ is a vector whose ith element is 1 and its other elements are zero, and the subindices o and p refer respectively, to offspring and parent. With probability ν_s a mutation in strategy occurs in which case a randomly chosen entry of the strategy matrix of the offspring is set randomly equal to either C or D.

We have considered different game structures. In the next section we start with a prisoner's dilemma (PD) game. Then we turn to another game frequently appealed in studying the evolution of cooperation. Namely, snow drift (SD), also known as hawk-dove and chicken game. The payoff of the games considered in this study are presented in Table. (SI.1).

	R	S	T	P		R	S	T	P		R	S	T	P
PD	0.6	0	0.8	0.2	TTD	0.6	0	1.4	0.2	SD	0.6	0.4	0.8	0.2
BS	0.4	0.8	0.6	0.2	leader	0.4	0.6	0.8	0.2					

Table SI.1. Games and their payoffs. R is the payoff to mutual cooperation, T payoff to defection against a cooperator, S payoff to cooperation with a defector, and P the payoff of mutual defection.

SI. 2 Mean field theory of the model

In this section we develop a mean field theory for the model. We proceed to write down expressions for the fitness of signals and strategies and use a selection-mutation equation to write a mean field equation for the model. In order to write down a mean field equation for the model, we use some simplifying assumptions. We assume individuals' signal is fixed during their life time. This simplified model can be of interest in many biological situations in which individuals' signal is a phenotype which is fixed during the life time. We note that this corresponds to the case $d\sigma = 1$ in our model. A second simplifying assumption is that, mutations in signal production and strategies occur as a result of agents randomly switching their signal and strategy. This is done, by assuming that each agent switches its signal to another signal chosen uniformly at random, with rate (probability per unit time) μ_σ. In addition, agents switch each entry of their strategy matrix to the opposite value (C to D and vice versa), with rate μ_s. Such an interpretation is more consistent with an interpretation of the evolutionary process as an imitation-exploration process, in which agents imitate available strategies and signals in the population proportional to their success, and at the same time, explore by randomly switching to novel strategies and signals. In this section we use lower case Latin letters (a, b, ...) to denote signals.

Consider the set of strategies which determine an individual's act if it shows signal a while its opponent shows signal b. That is $s(a,b)$. This can be either C or D. We show the density of strategies of the form $s(a,b)=C$ by $\rho_{a,b}$. Obviously, the density of strategies of the form $s(a,b)=D$ is equal to $1-\rho_{a,b}$. In addition, we show the density of signal a by ρ_a. This is the number of times signal a is produced in the population at a time step.

We begin by writing an expression for the fitness of signals. Consider the expected payoff of signal a (by this we mean the expected payoff accrued to an individual by showing signal a) against signal b. We show this by $w(a|b)$. Under the mean field assumption, this can be written as:

$$w(a|b)=R\rho_{a,b}\rho_{b,a}+S\rho_{a,b}(1-\rho_{b,a})+T(1-\rho_{a,b})\rho_{b,a}+P(1-\rho_{a,b})(1-\rho_{b,a}). \quad \text{(SI.1)}$$

Here, the first term is the probability that both signalers cooperate times the payoff of signal a in this case (R), the second term is the probability that a-signaler cooperates while the b-signaler defects times the payoff of signal a (S), the third term is the probability that a-signaler defects while the b-signaler cooperates times the payoff of signal a (T), and finally the last term is the probability that both signalers defect times the payoff of signal a (P). The expected payoff of signal a is equal to the average of $w(a|b)$ over b:

$$w(a)=\sum_b w(a|b)\rho_b. \quad \text{(SI.2)}$$

As mentioned above, signals are also subject to mutation. Taking into account both selection of signals based on their payoff and mutations in signals, the following mean field equation for the evolution of signal density can be written:

$$\frac{d\rho_a}{dt}=\rho_a[w(a)-c(a)-\langle w(a)-c(a)\rangle_a]-\mu_\sigma\rho_a+(1-\rho_a)\frac{\mu_\sigma}{n-1}. \quad \text{(SI.3)}$$

Here, $c(a)$ is the cost of signal a, and $\langle w(a)-c(a)\rangle_a=\sum_a\rho_a(w(a)-c(a))$, is the average net payoff (payoff minus cost) of signals. The first term ensures signal densities grow proportional to their fitness, the second term is the loss of density due to mutations out of a signal, and the last term is the gain in density due to mutations to the signal.

In the same way, we can write evolution equation for the density of strategies. The expected payoff of a strategy of the form $s(a,b)=C$ is:

$$w_C(a,b)=\rho_a\rho_b[R\rho_{b,a}+S(1-\rho_{b,a})]. \quad \text{(SI.4)}$$

The first term is the probability that an individual shows signal a, while its opponent shows signal b, times the probability that the opponent cooperates ($\rho_{b,a}$) times R (which is the payoff of the strategy $s_{a,b}=C$), and the second term is the probability that an individual shows signal a, while its opponent shows signal b, times the probability that the opponent defects $(1-\rho_{b,a})$ times S (which is the payoff of the strategy $s_{a,b}=C$). In the same way, the expected payoff of the complementary strategy $s(a,b)=D$ is:

$$w_D(a,b)=\rho_a\rho_b[T\rho_{b,a}+P(1-\rho_{b,a})]. \quad \text{(SI.5)}$$

In addition to growing proportional to their payoff, strategies are also subject to mutations. Taking both effects into account, the following mean field equation for the density of strategies can be written:

$$\frac{d\rho_{a,b}}{dt}=\rho_{a,b}(w_C(a,b)-[\rho_{a,b}w_C(a,b)+(1-\rho_{a,b})w_D(a,b)])-\mu_s\rho_{a,b}+(1-\rho_{a,b})\mu_s. \quad \text{(SI.6)}$$

The term in the bracket is the average payoff of strategies $s(a,b)=C$ and $s(a,b)=D$. The first term ensures that the density of each possible entry of the strategy matrix ($s(a,b)=C$ and $s(a,b)=D$) grows with a rate proportional to its payoff, the second term is the mutation out of a strategy, and the last term is the mutation to a strategy. Eq. (SI.3) and eq. (SI.6), provide a set of equations which can be simultaneously solved for all a and b, to yield evolution of the density of signals and strategies in the system.

In Fig. (SI.1), numerical solutions to the mean field equations are presented. Here, the prisoner's dilemma game, with payoff values given in Table. (SI.1) is considered, and the signal costs are randomly distributed in the interval $[0,c_{\max}]$, with $c_{\max}=0.1$. In Fig. (SI.1.a) and Fig. (SI.1.b), the densities of different strategy pairs CD (solid green line), CC (dash-dotted blue line), DD (dashed red line), for different mutation rates (indicated in the figure) are presented. For large mutation rates ($\geq 10^{-4}$) a large density of cooperative strategies is maintained in the system. The fraction of CD strategy pairs is close to 0.5, and the fraction of CC strategies increases with increasing mutation rate and approaches 0.25 for very large mutation rates. These features are consistent with agent based simulations of the model. The density of cooperative strategies however, decreases for

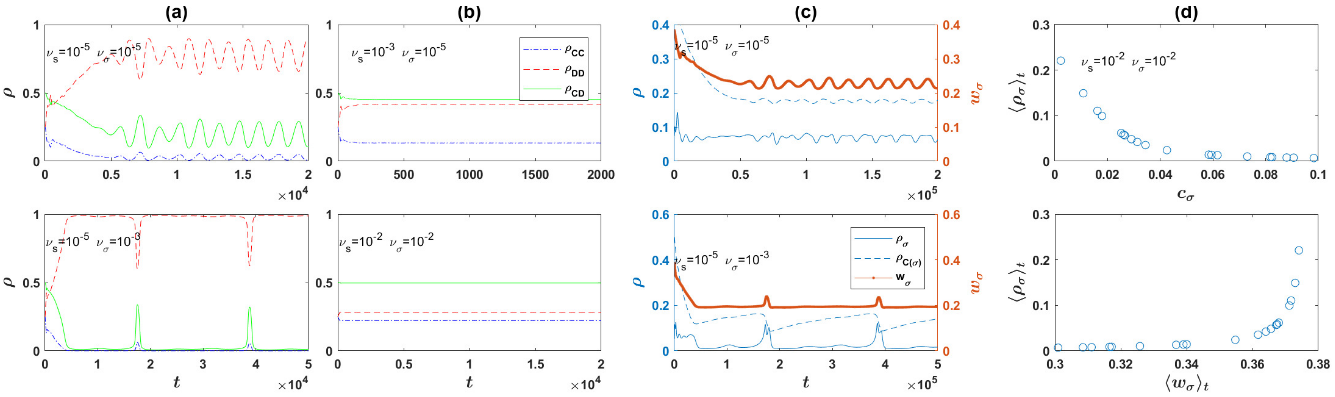

Figure SI.1. Numerical solutions of the mean field equations. (a) and (b): Fraction of strategy pairs as a function of time (Green solid line CD, red dashed line DD, blue dash-dotted line CC). In the mean field approximation, the level of cooperation increases with increasing the mutation rate in strategies ν_s. (c): Time evolution of density of a randomly chosen signal σ, density of the strategies which cooperate with that signal $C(\sigma)$, and fitness of that signal w_σ, for two different mutation rates indicated in the figures. The density of the strategies which cooperates with a signal increases when the density of that signal is low. When $\rho_{C(\sigma)}$ increases enough, the fitness of the signal increases, and consequently its density increases too. This decreases the fitness of strategies $C(\sigma)$, and thus $\rho_{C(\sigma)}$ decreases. The dynamics go through the same cycles. (d) Time average density of signals $\langle\rho_\sigma\rangle_t$ as a function of signal cost (up), and as a function of time average signal fitness $\langle w_\sigma\rangle_t$ (down). Mean field theory predicts a decreasing trend between signal cost and density, and an increasing trend between signal fitness and signal density. Here, the mean field equations are solved numerically for $t = 2000$ time steps and an average over the last 1000 time steps is taken.

very small mutation rates ($\leq 10^{-5}$). We have not observed such a decrease in agent based simulations for comparable mutation rates. This seems to suggest mean field theory deviates from the results of the dynamics for very small mutation rates. The reason is not difficult to see. In the mean field theory, correlations between different entries of the strategy matrix are neglected. As described below, these correlations play an important role in the dynamics of the system. When the density of a given signal b is low, strategies which cooperate with that signal (i.e. strategies of the form $s(a,b) = C$), impose small fitness cost and can grow and get fixed in the population due to association with fit strategies. This means that the same individual can have strategies of the form $s(a,b) = C$ and $s(a,b') = D$. When the density of the signal b is low while the density of b' is high, such individuals accumulate a high payoff and grow in number. This leads to an increase of cooperative strategies with the rare signal b. However, in the mean field assumption, such correlations are neglected and consequently, cooperative strategies can not be accumulated due to association with fit defective strategies. This leads to a reduction in the level of cooperative strategies in the mean field theory, compared to the agent based simulations.

In Fig. (SI.1.c), we take a look at the dynamics of signals and strategies in the mean field theory, by plotting the density of one of the signals ρ_σ and its fitness w_σ, together with the fraction of strategies which cooperate with that signal $\rho_{C(\sigma)}$, for two different mutation rates (indicated in the figure). The same dynamic observed in the agent based simulations, is grasped by the mean field theory. When the density of a signal is low, strategies which cooperate with that signal impose a small cost on their bearer. Consequently, the fraction of such strategies increase. This is observable in both cases presented in Fig. (SI.1.c), however, more salient in the lower panel of Fig. (SI.1.c), where the rate of mutation in strategies is much smaller than that of signals. When the density of cooperative strategies with the given signal increases enough, the signal reaches a high fitness. Consequently, its density increases. This in turn decreases the fitness of strategies which cooperate with that signal, and thus $\rho_{C(\sigma)}$ decreases. However, such a dynamics is observed only in small ν_s. When, ν_s increases, the mean field theory predicts signal and strategy densities to go to a fixed point and do not show variations in time. While according to the simulations, the same interrelated dynamics of signals and strategies observed in low mutation rates in the mean field theory, is at work for large mutation rates as well. This is another feature of the dynamics resulting from correlations between strategies, which is neglected in the mean field theory.

Finally, in Fig. (SI.1.d), we look at the relation between signal density, and cost, and also between signal density and fitness. As can be seen, the mean field theory predicts a non-zero density for costly signals. This results from the fact that costly signals receive a high payoff in the course of dynamics of the system which compensate for their cost. More precisely, as the density of costly signals is smaller compared to less costly signals, the fraction of cooperative strategies with costlier signals is higher. This partially compensate for their cost, and leads to a non-zero abundance of costly signals. We note that, without such mechanism, the dynamics would have produced a stationary state in which the density of all the signals except the cheapest one

would have been zero. In addition, the mean field theory predicts signal density is a decreasing function of signal cost (upper panel in Fig. (SI.1.d)) and an increasing function of signal fitness (lower panel in Fig. (SI.1.d)). These features are consistent with agent based simulations of the model.

SI. 3 Dependence of cooperation level on model parameters

SI. 3.1 Prisoner's dilemma game

The dependence of the cooperation level on the model parameters in the prisoner's dilemma game is investigated in Fig. (SI.2). The upper part of each panel shows the average fraction of C (blue lines marked by circles) and D (red lines marked by squares) strategies in the population. The lower part of each panel shows the fraction of strategy pairs actually played in the population. These include mutual cooperation CC (blue circles), mutual defection (red squares), and heterogeneous cooperation-defection CD (green triangles). The base parameter values are given in Table. (SI.2). These are the same parameter values used in the simulations in the main text. In each simulation, one of the parameters of the model is varied as specified in the figure. The fraction of strategies are calculated from a time series of the system of length $t = 50000$ after discarding the first $t = 1000$ steps.

parameter	ν_s	ν_σ	$d\sigma$	$c_{\max}$	n	N	
value	0.05	0.05	0.1	0.1	20	400	

Table SI.2. Base parameter values used in the simulations.

The dependence on the temptation T is investigated in Fig. (SI.2.a). Here, T is varied from 0.5 to 2. As R is fixed at 0.6, the prisoner's dilemma includes Ts in the interval $[0.6, 1.2]$. In the upper part of Fig. (SI.2.a), we see that, by increasing T, the fraction of C strategies decreases, and the fraction of D strategies increases. For small Ts the fraction of C strategies is larger, and at a certain T value in the PD range, the fraction of D strategies becomes larger. More interesting is how the fraction of strategy pairs actually played in the population changes. As can be seen in the lower part of Fig. (SI.2.a), the fraction of CCs decreases with increasing T. For small Ts this reduction in the density of CC is accompanied by an increase in both the density of DD and CD pairs. However, for larger Ts, the density of DDs become fixed while the density of CDs continues to increase well into the turn taking dilemma regime (i.e. when the prisoner's dilemma becomes a turn taking dilemma which happens at $T \geq 2R = 1.2$). In the turn taking dilemma range, the heterogeneous CD pair is in fact the socially optimal solution to the game. Thus as we can see, signaling can be a natural way to solve the cooperation dilemma and provides a social optimal solution, in the regime (i.e. turn taking dilemma) where, many of the conventional known solutions of the cooperation dilemma fail to solve the dilemma (As conventional solutions can only give rise to homogeneous CC strategies).

The dependence on the number of signals n is investigated in Fig. (SI.2.b). We see that the fraction of C strategies (upper part of Fig. (SI.2.b)), and also the fraction of CC and CD strategy pairs (lower part of Fig. (SI.2.b)) rapidly increases with increasing n up to $n = 20$. For larger n, although still increasing, but with a slower rate. This shows that signaling is more efficient in resolving the social dilemma in the PD when the number of signals is large enough.

In Fig. (SI.2.c) the dependence of the cooperation level on the maximum cost of signals c_{max} is investigated. For $c_{max} = 0$ signals have no cost. We see that up to moderate cost $c_{max} = 0.2$, cost of signals does not affect the level of cooperation adversely. An absolute cost of $dc = 0.2$ coincides to a normalized cost (absolute cost divided by mean payoff of the individuals from the game) of approximately 0.5. This means agents can invest up to half of their payoff in growing and maintaining costly signals. However, for larger costs, it becomes less profitable for agents to produce costly signals. Consequently, agents resort less to signaling as a way to solve the dilemma. Signal distributions become more homogeneous (maximized around the cheapest signals) and the level of competition between agents to maximize their payoff by engaging in a *signal war* decreases. Consequently, the level of D and mutual defection DD increase.

The dependence on the population size N is investigated in Fig. (SI.2.d). We see that for small population sizes, by increasing the population size the fraction of C strategies decreases while the fraction of D strategies increases. This reduction leads to a reduction of mutual cooperation while the fraction of CD strategies shows small sensitivity to the population size. For larger populations, the sensitivity of the density of strategies to the population size decreases.

The dependence on the mutation rate in strategies is investigated in Fig. (SI.2.e). Both the fraction of cooperation and mutual cooperation decrease by increasing ν_s for small mutation rates. The sensitivity to the mutation rate is stronger for small mutation rate and significantly decreases for large mutation rates.

The dependence on the mutation rate in signals is investigated in Fig. (SI.2.f). Here, the cooperation level changes depending on whether the mutation rate in signals is smaller or larger than the mutation rate in strategies. Both ρ_C and ρ_{CC} are maximized when ν_σ is comparable to ν_s. In other words, the level of cooperation is maximized when the time scale of evolution of signals and strategies are comparable and a gap in the time scale of the change of strategies and signals undermines

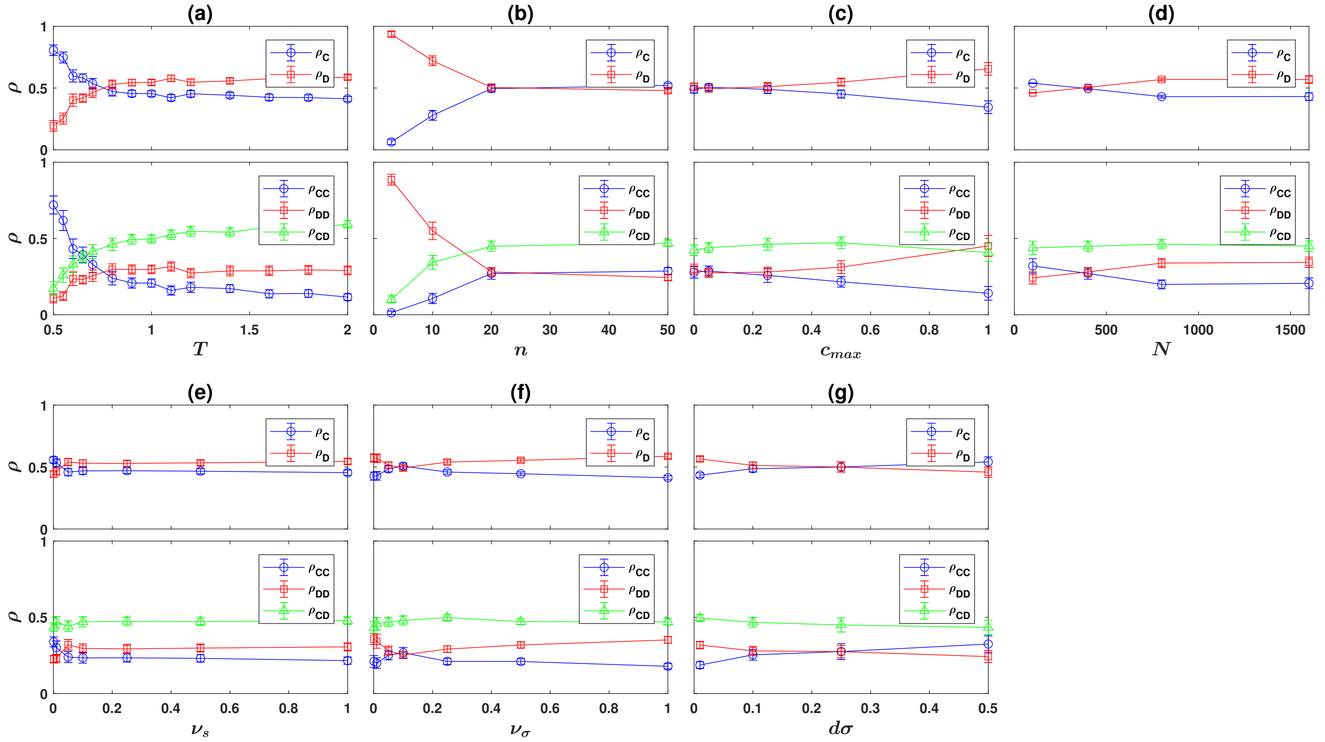

Figure SI.2. Parameter dependence of cooperation level in the prisoner's dilemma game. In each panel the dependence on one of the parameters is investigated. T is the temptation, n is the number of signals, c_{max} is the maximum cost of signals. N is the population size, ν_σ is the rate of mutation is signals, ν_s is the rate of mutation in strategies, and $d\sigma$ is the strength of mutations in signals. The upper part of each panel shows the fraction of C (cooperation) and D (defection) in the strategy matrix of individuals, averaged over population. The lower panel shows the fraction of strategy pairs actually played in the population. CC refers to mutual cooperation, DD to mutual defection, and CD refers to the case that an individual cooperates while the opponent defects. The simulations are run for $t = 50000$ time steps and an average is taken after discarding the first $t = 1000$ time steps.

cooperation. When $\nu_\sigma \ll \nu_s$, strategy densities become fast changing variables which adapt to the slowly changing signal distributions in the population. Such adaptation undermines cooperation, as defective strategies with high fitness are produced more often and grow in number by selection. In the opposite regime, when $\nu_s \ll \nu_\sigma$, agents have more chances to exploit cooperators by changing the probability with which they produce signals. In other words, strategies being a slowly changing variable with respect to the signal production probability, agents have the chance to exploit cooperators by changing their signal production probabilities in a way such that they get the most exploitation out of cooperators. This reduces cooperators' payoff the thus, decreases the frequency of cooperative strategies and mutual cooperation. To summarize, when there is a large gap between time scale of change of strategies and signals, agents can use this gap, either to exploit the slowly changing profile of strategies by producing fit signals (when $\nu_s \ll \nu_\sigma$), or to switch to fitter strategies which defect more often against the prevalent signals (when $\nu_\sigma \ll \nu_s$). Both effects undermine cooperation, and the cooperation level is maximized when the time scale of change of signals and strategies are comparable.

Dependence on the strength of mutations in signals is investigated in Fig. (SI.2.g). In the upper part of Fig. (SI.2.g), we see that, by increasing $d\sigma$, the fraction of cooperative strategies C increases, while the fraction of D decreases. As can be seen in in the lower part of Fig. (SI.2.g), this increase in the fraction of C comes with increasing the fraction of mutual cooperation, while the fraction of both mutual defection and CD strategy pairs decrease with increasing $d\sigma$.

SI. 3.2 Snow drift game

Another game frequently appealed to in studding the evolution of cooperation is the snow drift (SD) game [18]. Contrary to the PD, in SD, a certain fraction of cooperation is maintained in the equilibrium in a well-mixed population. For this reason, the evolution of cooperation in this game is not as a serious problem as in PD. Consequently, SD has attracted less attention compared to PD in studies of evolution of cooperation. However, the equilibrium level of cooperation in the SD

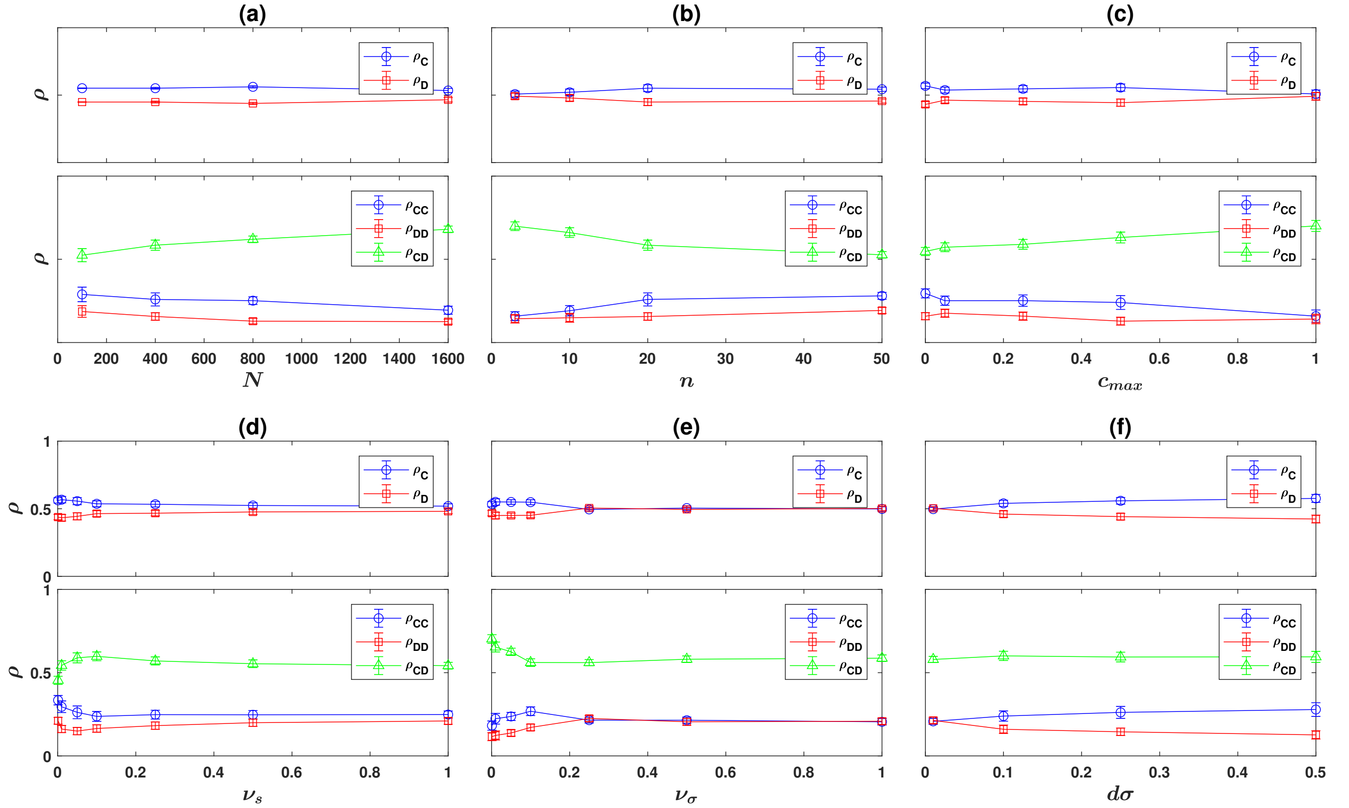

Figure SI.3. Parameter dependence of cooperation level in the snow drift game. In each panel the dependence on one of the parameters is investigated. N is the population size, n is the number of signals, c_{max} is the maximum cost of signals, ν_σ is the rate of mutation is signals, ν_s is the rate of mutation in strategies, and $d\sigma$ is the strength of mutations in signals. The upper part of each panel shows the fraction of C (cooperation) and D (defection) in the strategy matrix of individuals, averaged over the population. The lower panel shows the fraction of strategy pairs actually played in the population. CC refers to mutual cooperation, DD to mutual defection, and CD refers to the case when an individual cooperates while the opponent defect. The simulations are run for $t = 50000$ time steps and an average is taken after discarding the first $t = 1000$ time steps.

game is smaller than the social optimum. For this reason, this game, just as the PD game, offers a social dilemma, and the mechanisms by which the level of cooperation can be increased in this game has been studied [18]. Many of the mechanisms, resolve the social dilemma in SD by promoting mutual cooperation. However, as both mutual cooperation and heterogeneous cooperation-defection strategy pairs lead to the same social payoff in the SD, mechanisms which allow the individuals to coordinate in heterogeneous strategy pairs can be as efficient as mutual cooperation in resolving the social dilemma in SD. Some mechanisms which accommodate this by breaking the symmetry between the players are identified. For example if individuals hold a territory, a strategy which defects if the owner of the territory, and cooperate otherwise, can lead to a social optimum solution of the SD game [18]. Our study shows signaling can be appealed too as a fundamental mechanism to resolve or reduce the social dilemma in SD by promoting heterogeneous cooperation-defection pairs. Such heterogeneous strategies offer a situation as efficient as homogeneous cooperation in the SD game. Below, we investigate the parameter dependence of cooperation and partial cooperation level in the SD game.

The dependence of the density of cooperative strategies ρ_C, and the defective strategies, ρ_D on the population size is shown in the top panel of Fig. (SI.3.a). As can be seen, both the densities of cooperation and defection are close to $\frac{1}{2}$, with the latter slightly larger than the former, for all population sizes. Comparison with PD (in Fig. (SI.2.d)) shows the level of cooperation is higher in SD compared to PD. The densities of the strategy pairs actually played in the population are shown in the lower panel of Fig. (SI.3.a). While both the densities of DD and CC strategy pairs decrease with increasing population size, the density of the heterogeneous CD strategy increases with population size. Furthermore, the density of CC strategy pair is larger than that of DD in all the population sizes. These features show that signaling is an efficient mechanism to resolve the social dilemma of cooperation in the SD game, specially in larger population sizes.

The dependence on the number of signals n is investigated in Fig. (SI.3.b). In the top panel ρ_C and ρ_D are plotted. While

for any number of signals both ρ_C and ρ_D are close to $\frac{1}{2}$, the density of cooperative strategies slightly increases with n. To see what effect this has on the density of strategy pairs played in the population, in the lower panel of Fig. (SI.3.b), we plot the density of strategy pairs as a function of n. As can be seen, the density of heterogeneous CD strategy pair decreases by increasing n, specially for small values of n. This is accompanied by an increase in the density of mutual cooperation (CC). The variation of the density of strategy pairs with respect to n is very small for larger values of n.

We investigate the dependence of the density of strategies on the maximum signal cost c_{max}, in Fig. (SI.3.c). As can be seen in the top panel, ρ_C and ρ_D are close to $\frac{1}{2}$ for all c_{max}, and show small variation with respect to c_{max}. However, for very large c_{max}, a slight decrease in the density of cooperative strategies is visible. By turning to the density of strategy pairs in the lower panel of Fig. (SI.3.c), we see that by increasing signal cost, the density of heterogeneous strategies increases, while both ρ_{CC} and ρ_{DD} decrease. This shows that, in the SD game, individuals can induce their opponent to cooperation when the cost of signals is higher. This was not the case in the PD game as can be seen by comparison of Fig. (SI.2.c) for PD and Fig. (SI.3.c) for SD.

In Fig. (SI.3.d) and Fig. (SI.3.e), the dependence on, respectively, the mutation rate in strategies ν_s, and the mutation rate in signal production ν_σ is investigated. In the top panels, small variation with respect to ν_s in both ρ_C and ρ_D is seen. The behavior of ρ_{CC}, seems to be similar to the PD game: it decreases with increasing ν_s (Fig. (SI.3.d)), and is maximized when ν_σ is comparable with ν_s (Fig. (SI.3.e)).

Finally, the dependence on the strength of mutations $d\sigma$ is investigated in Fig. (SI.3.f). As can be seen in the top panel, the density of cooperative strategies, ρ_C increases, while ρ_D decreases, by increasing $d\sigma$. In the lower panel, the dependence of strategy pairs played in the population, as a function of $d\sigma$ is plotted. Here, we see that ρ_{CD} shows small variation with respect to $d\sigma$. On the other hand, the density of mutual cooperation increases, and the density of mutual defection decreases by increasing $d\sigma$.

SI. 4 Dependence of signal frequency on cost, and on fitness for different values of model parameters

In this section we systematically investigate the dependence of signal density on fitness, and on apparent cost for different values of the parameters of the model. We show that in all the cases (all the games and all values of model parameters) time average of signal density shows strong dependence on, and is an increasing function of, time average signal payoff. While the dependence of time average signal density on the apparent cost of signals is much weaker, and in many cases it is not possible to establish a trend between the two. We begin by the prisoner's dilemma game, and then proceed to other games.

SI. 4.1 Prisoner's dilemma game

To show that signal fitness determines signal densities for all parameter values, we run simulations with the base parameter values given in Table. (SI.2). However, in each simulation we change one of the parameters to test whether the trend between signal density and signal fitness, found in the main text, is valid in all parameter regions. The result is given in Fig. (SI.4) for the PD game. In each panel, one of the parameters is changed and time average signal density $\langle\rho_\sigma\rangle_t$ as a function of average of the payoff gathered by that signal $\langle w^a_\sigma\rangle_t$, over the same time window is plotted (this can be considered as the realized fitness of the signals and in the following is called realized fitness, or fitness of signals). In the inset of each panel, time average signal density as a function of normalized apparent cost (defined as apparent cost divided by average payoff of the individuals received from the games) is plotted too. In Fig. (SI.4.a) and Fig. (SI.4.b), the mutation rate in strategies is set equal to respectively $\nu_s = 0.001$ and $\nu_s = 0.5$. In Fig. (SI.4.c) and Fig. (SI.4.d), the mutation rate in signal is set equal to respectively $\nu_\sigma = 0.001$ and $\nu_\sigma = 0.5$. In Fig. (SI.4.e) and Fig. (SI.4.f), maximum cost is set equal to respectively $c_{max} = 0$ and $c_{max} = 1$. In Fig. (SI.4.g) and Fig. (SI.4.h), the strength of mutation in signals is set equal to respectively $d\sigma = 0.01$ and $d\sigma = 0.5$. In Fig. (SI.4.i) and Fig. (SI.4.j), the number of signals is set equal to respectively $n = 5$ and $n = 50$. And finally, In Fig. (SI.5.a) and Fig. (SI.4.b), the population size is set equal to respectively $N = 100$ and $N = 1600$. In all the cases the time window over which an average is taken is set equal to 15000 (except the last case in which $t = 10000$). Variation of time window length does not affect the results and our conclusions are valid for other time durations as well (see below).

In all the cases, when time average signal density as a function of time average payoff of signals is plotted, a strong increasing trend is observable. This trend results from the fact that signals are *indirectly* subject to selection based on their fitness. Consequently, signals with larger fitness are selected more often and assume a higher density in the population. On the other hand, when the time average density of signals as a function of signal cost is plotted (insets), in many cases no trend is observed. For some parameter values a weak decreasing trend is observed. However, in all the cases, the trend between signal density and signal fitness is (much) stronger than that between signal density and signal cost (This is shown more quantitatively below).

Of particular interest are Fig. (SI.4.e) and Fig. (SI.4.f), where the dependence on maximum cost is investigated. In Fig. (SI.4.e), signals have no cost. We see that our theory is valid in the case of costless signals as well and signal densities are

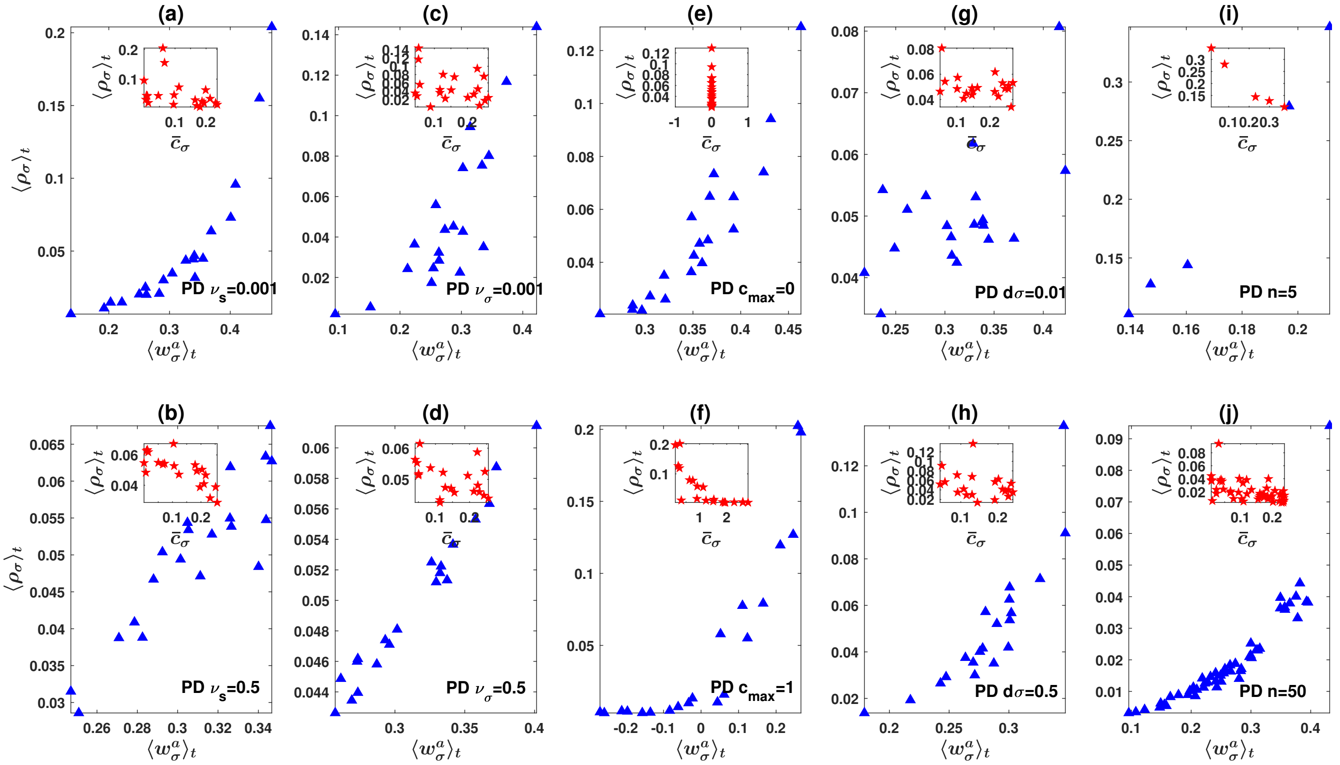

Figure SI.4. Investigation of the trend between signal frequency and signal fitness for different parameter values in the prisoner's dilemma game. Time average density of signals $\langle \rho_\sigma \rangle_t$ as a function of the time average realized fitness of the signals $\langle w^a_\sigma \rangle_t$ is plotted for different parameter values (different panels). The base parameter values are given in Table. (SI.2). In order to investigate the parameter dependence, in each panel, one of the parameters is changed (as specified in the figure). As can be seen, in all the cases $\langle \rho_\sigma \rangle_t$ shows an increasing trend with respect to $\langle w^a_\sigma \rangle_t$. For each panel, $\langle \rho_\sigma \rangle_t$ as a function of normalized apparent cost of the signals $\bar{c}_\sigma$ is shown in the corresponding inset. For some parameter values no trend between $\langle \rho_\sigma \rangle_t$ and $\bar{c}_\sigma$ is seen, and in all the cases the trend between $\langle \rho_\sigma \rangle_t$ and $\langle w^a_\sigma \rangle_t$ is much stronger than the trend between $\langle \rho_\sigma \rangle_t$ and $\bar{c}_\sigma$. Here, the time average is taken over a time window of length $t = 15000$, starting from the beginning of the simulations.

determined by signal fitness in this case too. In Fig. (SI.4.f), maximum signal cost is $c_{max} = 1$. As signals are distributed randomly in the interval $[0, c_{max}]$, most of the signals have extravagant cost in this case. In fact, noting that the maximum payoff reached in the game can be $T = 0.8$, signal costs can be larger than agent's payoff. This is seen in the inset of Fig. (SI.4.f), where normalized cost of signals can be close to 3 (i.e. signal cost can be three times agent's average payoff from the game). Obviously, no agent affords to produce such heavily costly signals. Consequently, for excessively costly signals, the density of signals become zero or close to zero.

We put the parameter dependence of the existence or lack thereof of a trend between signal density and fitness, (and between signal density and cost) to a more quantitative test as well. For this purpose we appeal to two statistical tests to examine the presence of a trend: Spearman's rank correlation test and Mann-Kendal trend test [57,58]. The results of the tests are given in Table. (SI.3). Here, the Spearman's rank correlation coefficient between time average signal density and normalized apparent cost $r(\bar{c}, \langle \rho_\sigma \rangle_t)$, and also Spearman's rank correlation coefficient between $\langle \rho_\sigma \rangle_t$ and time average signal realized fitness $\langle w^a_\sigma \rangle_t$, denoted as $r(\langle w^a_\sigma \rangle_t, \langle \rho_\sigma \rangle_t)$, are given in first rows. The p value of the Spearman's test p_r, and also the p value of the Mann-Kendall test p_{MK} are also given. A p value smaller than 0.05 is conventionally considered as a success in establishing a trend and a p value larger than 0.05 is considered as a failure in establishing the trend. For each of the model parameters, 3 different values are considered and r, p_r, and p_{MK} (each first for the normalized cost and time average density, and then for time average fitness and time average density) are presented. As can be seen, in most of the cases both tests fail to establish a trend between time average density and normalized apparent costs. While a strong or a very strong trend between time average density and time average fitness of signals is established using both tests. In addition, in all the cases, the (increasing) trend between time average density and time average fitness is (much) stronger than the (decreasing) trend between time average density and normalized cost of signals. This means that, signal fitness explains signal densities in the population, much better than the signal costs, and can be seen as a solution to the puzzle posed by the presence of costly signals.

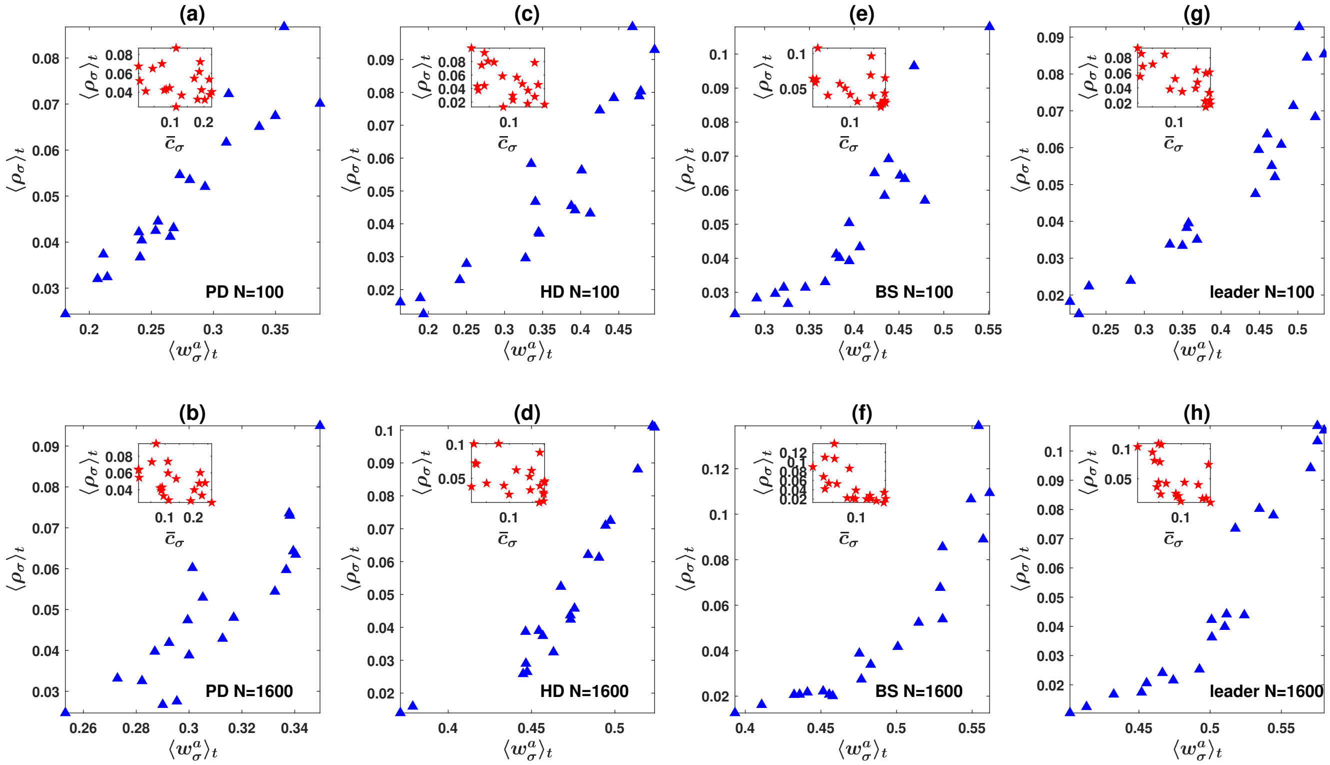

Figure SI.5. Dependence of the trend between signal frequency and signal fitness on population size in all the games considered. Time average density of signals $\langle \rho_\sigma \rangle_t$ as a function of the time average realized fitness of the signals $\langle w_\sigma^a \rangle_t$ is plotted for two different population sizes, for different games (different panels, as specified in the figure). The base parameter values are given in Table. (SI.2). As can be seen, in all the cases $\langle \rho_\sigma \rangle_t$ shows an increasing trend with respect to $\langle w_\sigma^a \rangle_t$. For each panel, $\langle \rho_\sigma \rangle_t$ as a function of normalized apparent cost of the signals $\bar{c}_\sigma$ is shown in the corresponding inset. For some parameter values no trend between $\langle \rho_\sigma \rangle_t$ and $\bar{c}_\sigma$ is seen, and in all the cases the trend between $\langle \rho_\sigma \rangle_t$ and $\langle w_\sigma^a \rangle_t$ is much stronger than the trend between $\langle \rho_\sigma \rangle_t$ and $\bar{c}_\sigma$. Here, the time average is taken over a time window of $t = 10000$, starting from the beginning of the simulations.

The time window over which the average is taken differs for different parameters in Table. (SI.3). It is equal to 1000 for ν_s and c_{max} and N (the same for all the three values presented in the table), and equal to 5000 for ν_σ, and 10^4 for $d\sigma$. Variations of this time does not affect the overall result, as long as it is larger than the possible time lag between the signal density and signal fitness (as for the signal fitness to affect signal density we need to take an average over a time window larger than the time lag between the two). This time lag results from the fact that when a signal's fitness increases, it takes some time until enough mutations happen and grow such that the density of that signal increases as well (see Fig. (1) in the main text for an example of a time lag between signal fitness and signal density). In most of the parameter values considered here, this time lag is rather small (less than 100 time steps). However, for small $d\sigma$ this time lag can be larger, as it takes longer time for mutations in signal production to be accumulated enough for the signal densities to follow signal fitness. Generally by increasing the averaging time window, a stronger trend between density and fitness is observed.

SI. 4.2 Snow drift game

We repeat the same experiments as in the last section, this time for the snow drift game. The base parameter values used in the simulations are given in Table. (SI.2). However, in each simulation we change one of the parameters to test whether the trend found between signal density and signal fitness is valid in all parameter regions. The result is given in Fig. (SI.6) for the SD game. In each panel, one of the parameters is changed and the time average signal density $\langle \rho_\sigma \rangle_t$ as a function of the average of the payoff gathered by that signal over the same time window $\langle w_\sigma^a \rangle_t$, is plotted. In the inset of each panel, time average signal density as a function of normalized apparent cost (defined as apparent cost divided by average payoff of the individuals from the games) is plotted too. In Fig. (SI.6.a) and Fig. (SI.6.b), the mutation rate in strategies is set equal to, respectively, $\nu_s = 0.001$ and $\nu_s = 0.5$. In Fig. (SI.6.c) and Fig. (SI.6.d), the mutation rate in signals is set equal to, respectively, $\nu_\sigma = 0.001$ and $\nu_\sigma = 0.5$. In Fig. (SI.6.e) and Fig. (SI.6.f), maximum cost is set equal to, respectively, $c_{max} = 0$ and $c_{max} = 1$.

v_s	0.001	0.05	1	v_σ	0.001	0.05	1
$r(\bar{c}_\sigma, \langle\rho_\sigma\rangle_t)$	-0.22	-0.20	-0.33		-0.24	-0.28	-0.45
$r(\langle w^a_\sigma\rangle_t, \langle\rho_\sigma\rangle_t)$	0.87	0.96	0.63		0.66	0.91	0.98
$p_r(\bar{c}_\sigma, \langle\rho_\sigma\rangle_t)$	3.3×10^{-1}	3.8×10^{-1}	1.5×10^{-1}		3.0×10^{-1}	2.1×10^{-1}	4.3×10^{-2}
$p_r(\langle w^a_\sigma\rangle_t, \langle\rho_\sigma\rangle_t)$	3.5×10^{-7}	8.6×10^{-12}	2.9×10^{-3}		1.3×10^{-3}	1.4×10^{-8}	8.2×10^{-15}
$p_{MK}(\bar{c}_\sigma, \langle\rho_\sigma\rangle_t)$	0.25	0.49	0.16		3.8×10^{-1}	1.6×10^{-1}	5.5×10^{-2}
$p_{MK}(\langle w^a_\sigma\rangle_t, \langle\rho_\sigma\rangle_t)$	1.1×10^{-5}	1.7×10^{-7}	5.8×10^{-3}		1.6×10^{-3}	4.7×10^{-6}	1.3×10^{-8}

dc	0.05	0.25	0.5	$d\sigma$	0.01	0.1	0.5
$r(\bar{c}_\sigma, \langle\rho_\sigma\rangle_t)$	-0.15	-0.60	-0.65		-0.07	-0.50	-0.22
$r(\langle w^a_\sigma\rangle_t, \langle\rho_\sigma\rangle_t)$	0.93	0.93	0.84		0.48	0.92	0.88
$p_r(\bar{c}_\sigma, \langle\rho_\sigma\rangle_t)$	5.0×10^{-1}	4.7×10^{-2}	1.6×10^{-3}		7.3×10^{-1}	2.1×10^{-2}	3.3×10^{-1}
$p_r(\langle w^a_\sigma\rangle_t, \langle\rho_\sigma\rangle_t)$	2.6×10^{-9}	5.5×10^{-9}	7.9×10^{-5}		3.0×10^{-2}	3.2×10^{-9}	2.5×10^{-7}
$p_{MK}(\bar{c}_\sigma, \langle\rho_\sigma\rangle_t)$	4.9×10^{-1}	1.0×10^{-2}	4.7×10^{-3}		7.2×10^{-1}	6.4×10^{-2}	2.2×10^{-1}
$p_{MK}(\langle w^a_\sigma\rangle_t, \langle\rho_\sigma\rangle_t)$	1.8×10^{-6}	6.9×10^{-7}	4.7×10^{-6}		2.1×10^{-2}	6.9×10^{-7}	3.4×10^{-6}

n	5	20	50	N	100	400	1600
$r(\bar{c}_\sigma, \langle\rho_\sigma\rangle_t)$	-0.70	-0.02	-0.45		-0.07	-0.11	-0.23
$r(\langle w^a_\sigma\rangle_t, \langle\rho_\sigma\rangle_t)$	1	0.84	0.92		0.94	0.83	0.88
$p_r(\bar{c}_\sigma, \langle\rho_\sigma\rangle_t)$	1.8×10^{-1}	9.0×10^{-1}	9.0×10^{-4}		7.4×10^{-1}	6.2×10^{-1}	3.1×10^{-1}
$p_r(\langle w^a_\sigma\rangle_t, \langle\rho_\sigma\rangle_t)$	0	2.5×10^{-6}	0		3.1×10^{-10}	4.7×10^{-6}	2.5×10^{-7}
$p_{MK}(\bar{c}_\sigma, \langle\rho_\sigma\rangle_t)$	2.2×10^{-1}	9.7×10^{-1}	2.0×10^{-3}		8.7×10^{-1}	5.3×10^{-1}	3.1×10^{-1}
$p_{MK}(\langle w^a_\sigma\rangle_t, \langle\rho_\sigma\rangle_t)$	2.7×10^{-2}	3.7×10^{-5}	9.5×10^{-15}		6.9×10^{-7}	5.0×10^{-5}	8.7×10^{-6}

Table SI.3. Trend test between time average density of signals $\langle\rho_\sigma\rangle_t$, and normalized apparent cost $\bar{c}$, and between $\langle\rho_\sigma\rangle_t$ and time average payoff of signals $\langle w^a_\sigma\rangle_t$ for different parameter values in the prisoner's dilemma game. From top to down, Spearman's rank correlation coefficient between $\langle\rho_\sigma\rangle_t$ and $\bar{c}$, and between $\langle\rho_\sigma\rangle_t$ and $\langle w^a_\sigma\rangle_t$, p value of the Spearman test between $\langle\rho_\sigma\rangle_t$ and $\bar{c}$, and between $\langle\rho_\sigma\rangle_t$ and $\langle w^a_\sigma\rangle_t$,, and finally, p value of the Mann-Kendall test between $\langle\rho_\sigma\rangle_t$ and $\bar{c}$, and between $\langle\rho_\sigma\rangle_t$ and $\langle w^a_\sigma\rangle_t$. In all the cases, both tests strongly support a trend between fitness and density of signals, but in most of the cases, fail to establish a trend between the apparent cost and density of signals. In all the cases, the trend between density and realized fitness is significantly stronger compared to the trend between apparent cost and density.

In Fig. (SI.6.g) and Fig. (SI.6.h), the strength of mutation in signals is set equal to, respectively, $d\sigma = 0.01$ and $d\sigma = 0.5$. In Fig. (SI.6.i) and Fig. (SI.6.j), the number of signals is set equal to, respectively, $n = 5$ and $n = 50$. And finally, In Fig. (SI.5.c) and Fig. (SI.5.d), the population size is set equal to, respectively, $N = 100$ and $N = 1600$. In all the cases the time window over which an average is taken is set equal to $t = 5000$. Variation of the time window length does not affect the results and our conclusions are valid for other time durations as long as the time duration is larger than a possible time lag that can exist between signal density and signal fitness.

As in the case of the prisoner's dilemma game studied in the last section, in all the cases, when time average signal density as a function of time average payoff of signals is plotted, a strong increasing trend is observable. This trend results from the fact that signals are *indirectly* subject to selection based on their fitness. Consequently, signals with larger fitness are selected more often and assume a higher density in the population. On the other hand, when the time average density of signals as a function of signal cost is plotted (insets), in many cases no trend is observed. For some parameter values a weak decreasing trend is observed. However, in all the cases, the trend between signal density and signal fitness is (much) stronger than that between signal density and signal cost. This conclusion is also valid in the case of costless signals plotted in Fig. (SI.6.e). In this case, even though signals are symmetric in that they all are costless, in the course of dynamics, this symmetry breaks and at each time instant, some signals find higher fitness compared to others. Such signals are produced more often. This leads to the increasing trend between signal fitness and signal density observed here.

SI. 4.3 Battle of the sexes game

Here, we check that a strong increasing trend between signal fitness and signal density also exists for different parameter values in the case of BS game. We repeat the same experiments, this time for the BS game. The procedure is as before: We use the parameter values given in Table. (SI.2) as the base parameter values. However, in each simulation one of the parameters is changed to test whether the trend found between signal density and signal fitness is valid in all parameter regions. The result is

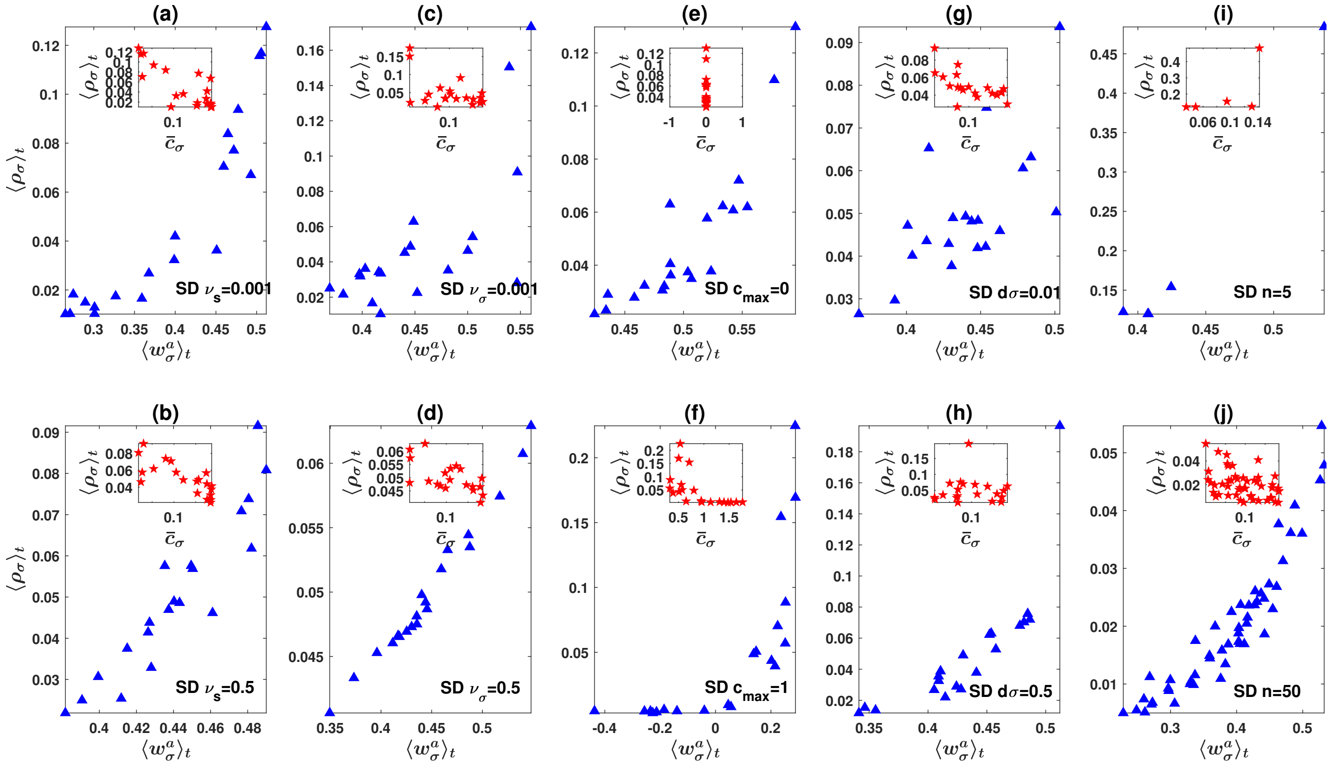

Figure SI.6. Investigation of the trend between signal frequency and signal fitness for different parameter values in the snow drift game. Time average density of signals $\langle \rho_\sigma \rangle_t$ as a function of the time average realized fitness of signals $\langle w_\sigma^a \rangle_t$ is plotted for different parameter values (different panels). The base parameter values are given in Table. (SI.2). In order to investigate the parameter dependence, in each panel, one of the parameters is changed (as specified in the figure). As can be seen, in all the cases $\langle \rho_\sigma \rangle_t$ shows an increasing trend with respect to $\langle w_\sigma^a \rangle_t$. For each panel, $\langle \rho_\sigma \rangle_t$ as a function of normalized apparent cost of the signals $\bar{c}_\sigma$ is shown in the corresponding inset. For some parameter values no trend between $\langle \rho_\sigma \rangle_t$ and $\bar{c}_\sigma$ is seen, and in all the cases the trend between $\langle \rho_\sigma \rangle_t$ and $\langle w_\sigma^a \rangle_t$ is much stronger than the trend between $\langle \rho_\sigma \rangle_t$ and $\bar{c}_\sigma$. Here, the time average is taken over a time window of $t = 5000$, starting from the beginning of the simulations.

given in Fig. (SI.7) for the BS game. In each panel, one of the parameters is changed and the time average signal density $\langle \rho_\sigma \rangle_t$ as a function of the average of the payoff gathered by that signal over the same time window $\langle w_\sigma^a \rangle_t$, is plotted. In the inset of each panel, time average signal density as a function of normalized apparent cost (defined as apparent cost divided by average payoff of the individuals from the games) is plotted too. In Fig. (SI.7.a) and Fig. (SI.7.b), the mutation rate in strategies is set equal to, respectively, $\nu_s = 0.001$ and $\nu_s = 0.5$. In Fig. (SI.7.c) and Fig. (SI.7.d), the mutation rate in signals is set equal to, respectively, $\nu_\sigma = 0.001$ and $\nu_\sigma = 0.5$. In Fig. (SI.7.e) and Fig. (SI.7.f), maximum cost is set equal to, respectively, $c_{max} = 0$ and $c_{max} = 1$. In Fig. (SI.7.g) and Fig. (SI.7.h), the strength of mutation in signals is set equal to respectively, $d\sigma = 0.01$ and $d\sigma = 0.5$. In Fig. (SI.7.i) and Fig. (SI.7.j), the number of signals is set equal to, respectively, $n = 5$ and $n = 50$. And finally, In Fig. (SI.5.e) and Fig. (SI.5.f), the population size is set equal to, respectively, $N = 100$ and $N = 1600$. In all the cases the time window over which an average is taken is set equal to $t = 5000$. Variation of the time window length does not affect the results and our conclusions are valid for other time durations as long as the time duration is larger than a possible time lag that can exist between signal density and signal fitness.

As in the case of the prisoner's dilemma game studied before, in all the cases, when the time average signal density as a function of time average payoff of signals is plotted, a strong increasing trend is observable. This trend results from the fact that signals are *indirectly* subject to selection based on their fitness. Consequently, signals with larger fitness are selected more often and assume a higher density in the population. On the other hand, when the time average density of signals as a function of signal cost is plotted (insets), in many cases no trend is observed. For some parameter values a weak decreasing trend is observed, however, in all the cases, the trend between signal density and signal fitness is (much) stronger than that between signal density and signal cost. This conclusion is also valid in the case of costless signals plotted in Fig. (SI.7.e). In this case, even though signals are symmetric in that they all are costless, in the course of dynamics, this symmetry breaks and at each time instant, some signals find higher fitness compared to others. Such signals are produced more often. This leads to the increasing

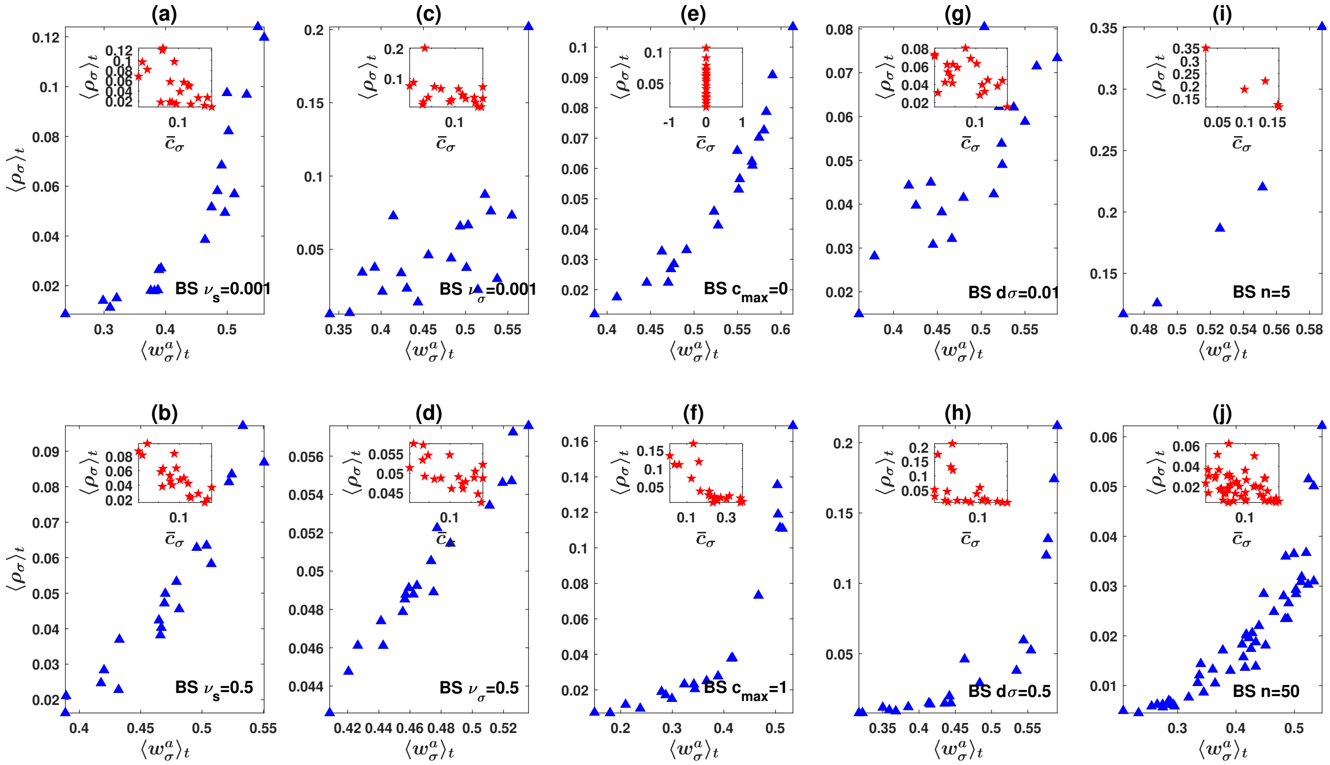

Figure SI.7. Investigation of the trend between signal frequency and signal fitness for different parameter values in the Battle of the sexes. Time average density of signals $\langle \rho_\sigma \rangle_t$ as a function of the time average realized fitness of the signals $\langle w^a_\sigma \rangle_t$ is plotted for different parameter values (different panels). The base parameter values are given in Table. (SI.2). In order to investigate the parameter dependence, in each panel, one of the parameters is changed (as specified in the figure). As can be seen, in all cases $\langle \rho_\sigma \rangle_t$ shows an increasing trend with respect to $\langle w^a_\sigma \rangle_t$. For each panel, $\langle \rho_\sigma \rangle_t$ as a function of normalized apparent cost of the signals $\bar{c}_\sigma$ is shown in the corresponding inset. For some parameter values no trend between $\langle \rho_\sigma \rangle_t$ and $\bar{c}_\sigma$ is seen, and in all the cases the trend between $\langle \rho_\sigma \rangle_t$ and $\langle w^a_\sigma \rangle_t$ is much stronger than the trend between $\langle \rho_\sigma \rangle_t$ and $\bar{c}_\sigma$. Here, the time average is taken over a time window of $t = 5000$, starting from the beginning of the simulations.

trend between signal fitness and signal density observed here.

SI. 4.4 Leader game

Finally, we check that the increasing trend between signal density and signal fitness holds for all parameter values also in the case of the leader game. As before, we use the base parameter values given in Table. (SI.2). However, in each simulation we change one of the parameters to test whether the trend found between signal density and signal fitness is valid in all parameter regions. The result is given in Fig. (SI.8) for the leader game. In each panel, one of the parameters is changed and time average signal density $\langle \rho_\sigma \rangle_t$ as a function of average of the payoff gathered by that signal $\langle w^a_\sigma \rangle_t$, over the same time window is plotted. In the inset of each panel, time average signal density as a function of normalized apparent cost (defined as apparent cost divided by average payoff of the individuals) is plotted too. In Fig. (SI.8.a) and Fig. (SI.8.b), the mutation rate in strategies is set equal to, respectively, $\nu_s = 0.001$ and $\nu_s = 0.5$. In Fig. (SI.8.c) and Fig. (SI.8.d), the mutation rate in signals is set equal to, respectively, $\nu_\sigma = 0.001$ and $\nu_\sigma = 0.5$. In Fig. (SI.7.e) and Fig. (SI.7.f), maximum cost is set equal to, respectively, $c_{max} = 0$ and $c_{max} = 1$. In Fig. (SI.8.g) and Fig. (SI.8.h), the strength of mutation in signals is set equal to, respectively, $d\sigma = 0.01$ and $d\sigma = 0.5$. In Fig. (SI.8.i) and Fig. (SI.8.j), the number of signals is set equal to, respectively, $n = 5$ and $n = 50$. And finally, In Fig. (SI.5.g) and Fig. (SI.5.h), the population size is set equal to, respectively, $N = 100$ and $N = 1600$. In all the cases the time window over which an average is taken is set equal to $t = 10000$. Variation of the time window length does not affect the results and our conclusion are valid for other time durations as long as the time duration is larger than a possible time lag that can exist between signal density and signal fitness.

As in the case of the prisoner's dilemma game, in all the cases, when time average signal density as a function of time average payoff of signals is plotted, a strong increasing trend is observable. On the other hand, when the time average density of signals as a function of signal cost is plotted (insets), in many cases no trend is observed. For some parameter values a weak

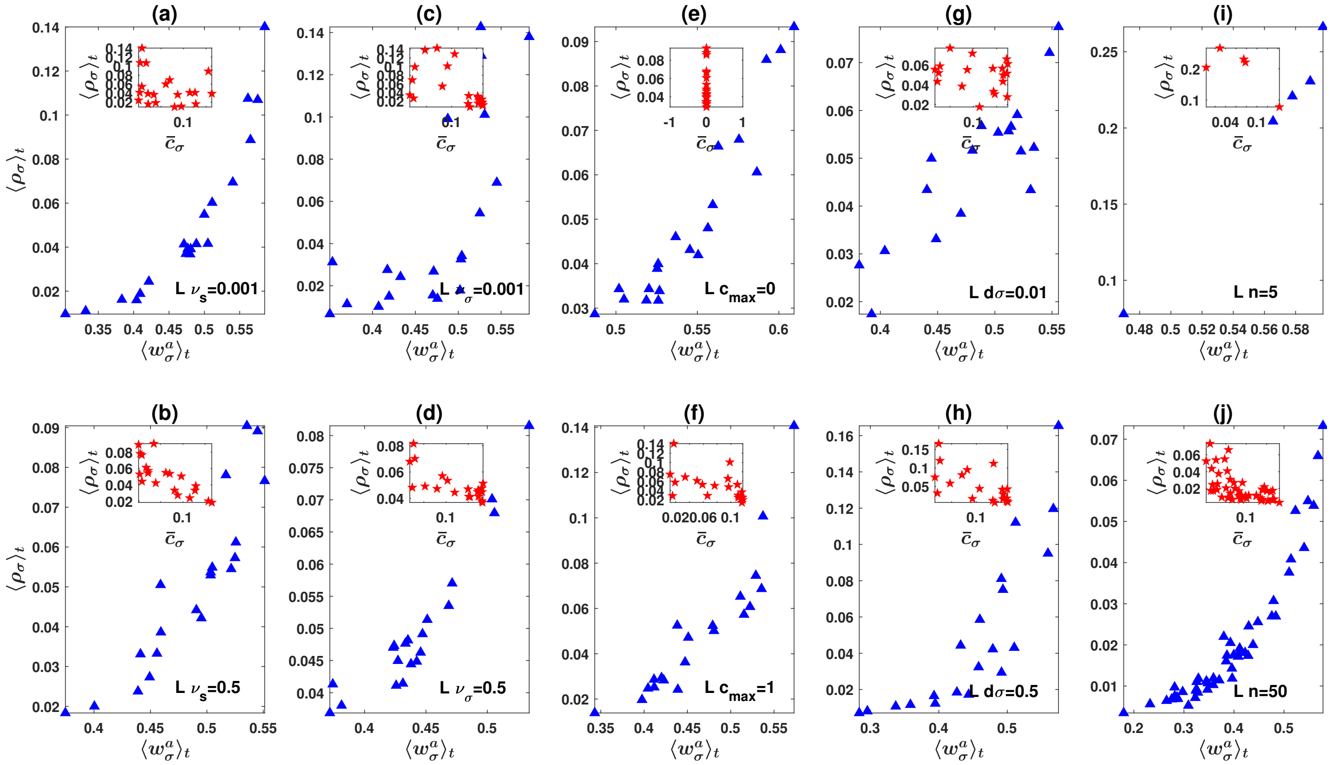

Figure SI.8. Investigation of the trend between signal frequency and signal fitness for different parameter values in the leader game. Time average density of signals $\langle \rho_\sigma \rangle_t$ as a function of the time average realized fitness of the signals $\langle w^a_\sigma \rangle_t$ is plotted for different parameter values (different panels). The base parameter values are given in Table. (SI.2). In order to investigate the parameter dependence, in each panel, one of the parameters is changed (as specified in the figure). As can be seen, in all cases $\langle \rho_\sigma \rangle_t$ shows an increasing trend with respect to $\langle w^a_\sigma \rangle_t$. For each panel, $\langle \rho_\sigma \rangle_t$ as a function of normalized apparent cost of the signals $\bar{c}_\sigma$ is shown in the corresponding inset. For some parameter values no trend between $\langle \rho_\sigma \rangle_t$ and $\bar{c}_\sigma$ is seen, and in all the cases the trend between $\langle \rho_\sigma \rangle_t$ and $\langle w^a_\sigma \rangle_t$ is much stronger than the trend between $\langle \rho_\sigma \rangle_t$ and $\bar{c}_\sigma$. Here, the time average is taken over a time window of $t = 10000$, starting from the beginning of the simulations.

decreasing trend is observed. However, in all the cases, the trend between signal density and signal fitness is (much) stronger than that between signal density and signal cost. This conclusion is also valid in case of costless signals plotted in Fig. (SI.7.e). In this case, even though signals are symmetric in that they all are costless, in the course of dynamics, this symmetry breaks and at each time instant, some signals find higher fitness compared to others. Such signals are produced more often. This leads to the increasing trend between signal fitness and signal density observed here.

SI. 5 When selection occurs with a probability proportional to the exponential of payoff

In this section, we consider a rather different model, in which selection occurs with a probability proportional to the exponential of payoff. All the details of this model are as before (as explained in the Model Section in this text). The only difference is that in the selection step of the evolutionary dynamics, agents are selected with a probability proportional to the exponential of their payoff. That is, agent α reproduce with a probability $\frac{\exp(\beta w_\alpha)}{\sum_\alpha \exp(\beta w_\alpha)}$. Here β can be considered as the strength of selection and determines the degree to which payoff differences determine reproductive success. As it has been noted in other models of evolution of cooperation, this seemingly innocent change can make important differences [27]. For example, while cooperation evolves on networks with a certain update rule in a model in which individuals reproduce with a probability proportional to the exponential of their payoff, but not if individuals reproduce with a probability proportional to their payoff.

Remarkably, in our model, no qualitative difference in the results is introduced when we make this change to the model: In a model in which individuals reproduce with a probability proportional to exponential of their payoff, cooperation and partial cooperation are evolved. Individuals compete to exploit the existent strategies by producing the signals which are more likely to be cooperated with. They also compete by changing their strategies to pay less cost of cooperation by being more defective with respect to the signals which are more frequent in the population. As a result of this signaling war, signals obtain a fitness due to

the internal dynamics of the system and the signal densities are determined by their fitness. The density of signals is explained better by the payoff they gather (i.e. the payoff an individual gathers by showing that signal), compared to their apparent cost. Below we look at this model in more detail. In the following, we will occasionally call this model the exponential model to distinguish it from the model considered before.

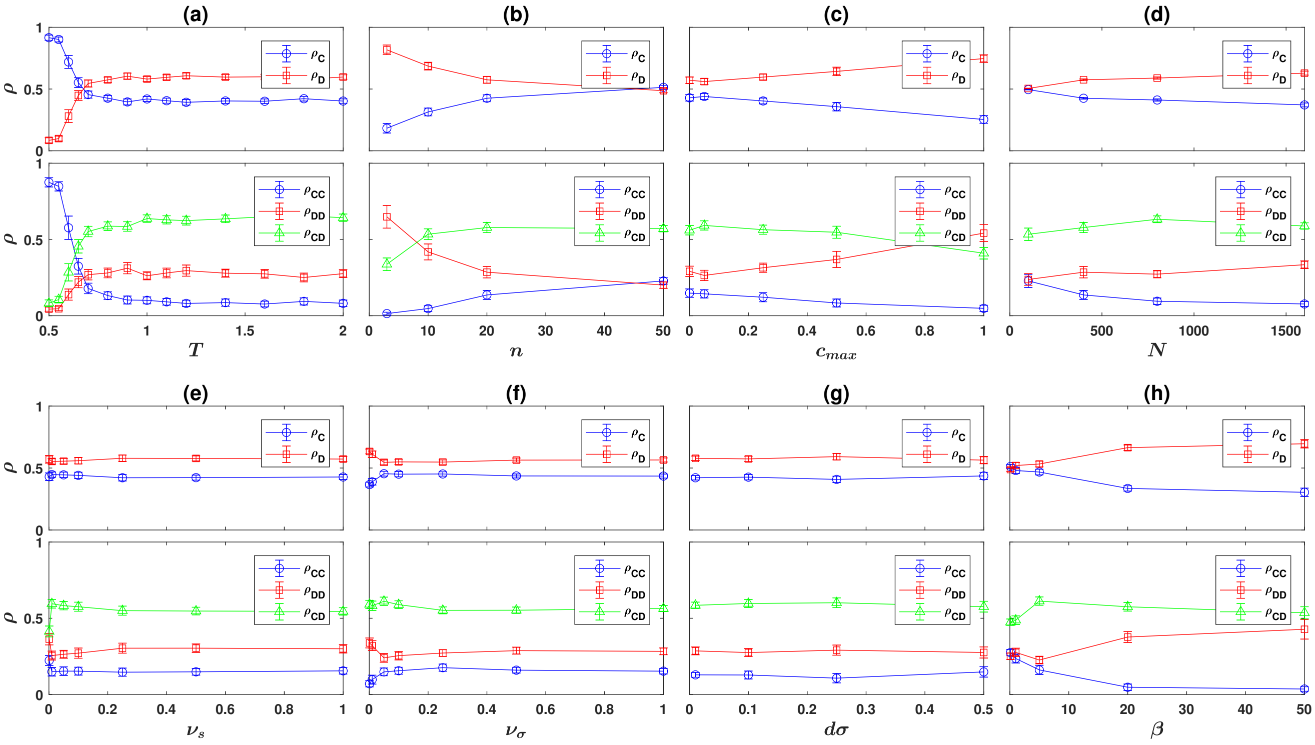

Figure SI.9. Parameter dependence of cooperation level in the prisoner's dilemma game, in the model in which individuals reproduce with a probability proportional to the exponential of their payoff. In each panel the dependence on one of the parameters is investigated. T is the temptation, n is the number of signals, c_{max} is the maximum cost of signals. N is the population size, ν_σ is the rate of mutation is signals, ν_s is the rate of mutation in strategies, and $d\sigma$ is the strength of mutations in signals. The upper part of each panel shows the fraction of C (cooperation) and D (defection) in the strategy matrix of individuals, averaged over population. The lower panel shows the fraction of strategy pairs actually played in the population. CC refers to mutual cooperation DD to mutual defection, and CD refers to the case that an individual cooperates while the opponent defects. Averages are taken on a time series of length $t = 50000$, after discarding the first 1000 time steps.

SI. 5.1 Dependence of the cooperation level on the model parameters in the exponential model

The dependence of the cooperation level on the model parameters, for the model in which individuals reproduce with a probability proportional to the exponential of their payoff, is investigated in Fig. (SI.9) for the prisoner's dilemma game. The upper part of each panel shows the average fraction of C (blue lines marked by circles) and D (red line marked by squares) strategies in the population. The lower part of each panel shows the fraction of strategy pairs actually played in the population. These include mutual cooperation CC (blue circles), mutual defection (red squares), and heterogeneous cooperation-defection strategy pairs CD (green triangles). The payoff values used in the simulations are the same as those given in Table. (SI.1) and the base parameter values are the same as those given in Table. (SI.2). In each simulation, one of the parameters of the model is varied as specified in the figure. The fraction of strategies are calculated from a time series of system of length $t = 50000$ after discarding the first $t = 1000$ steps.

The dependence on the temptation T is investigated in Fig. (SI.9.a). Here, T is varied from 0.5 to 2. As R is fixed at 0.6, the prisoner's dilemma includes Ts in the interval $[0.6, 1.2]$. We see that, by increasing T, the fraction of C strategies, and CC pairs decrease, and the fraction of D strategies increases. the fraction of both C and CC strategy pairs show a sharp decrease at a value $T = R = 0.6$. This is the value at which the game becomes a (prisoner's) dilemma. The reduction in the fraction of C strategies is accompanied by an increase in the fraction of D strategies (upper part of Fig. (SI.9.a)). While the reduction in the fraction of CC strategy pair is accompanied by an increase in both DD and CD strategy pairs (lower part of Fig.

(SI.9.a)). In the PD region, the fraction of CD strategies is the larges compared to the other two possible strategy pairs. This means CD strategy pair is the dominant strategy pair. This continues to hold well into the turn taking dilemma region ($T > 2R$), where the heterogeneous CD strategy pair becomes the social optimum (as it endows the largest total payoff compared to the homogeneous CC or DD strategy pairs). By comparison with the model in which individuals reproduce with a probability proportional to their payoff, presented in Fig. (SI.2.a), we see that the main quantitative difference is a slight reduction in mutual cooperation in the exponential model.

The dependence on the number of signals n is investigated in Fig. (SI.9.b). We see that the fraction of C strategies (upper part of Fig. (SI.9.b)), and also the fraction of CC and CD strategy pairs (lower part of Fig. (SI.9.b)) increase with increasing n. The main difference with the model in which reproduction is done with a probability proportional to payoff is that the rate of increase in cooperative strategies with increasing n is smaller in the exponential model.

In Fig. (SI.9.c), the dependence of the cooperation level on the maximum cost of signals is investigated. For $c_{max} = 0$ signals have no cost. We see that both the fraction of C strategies (upper part of Fig. (SI.9.c)) and the CC and CD strategy pairs (lower part of Fig. (SI.9.c)) decrease with increasing cost. This reduction in the level of cooperation is less rapid for small costs and becomes more rapid as cost of signals increases. The reason is that for larger cost, it pays less for the agents to engage in signaling wars by producing different costly signals and they stick more to homogeneous production of the cheapest signals, which are defected more often.

The dependence of the cooperation level on the population size N is investigated in Fig. (SI.9.d). We see that for small population sizes, by increasing the population size, keeping the number of signals n fixed, the fraction of C strategies decreases while the fraction of D strategies increase. This reduction in ρ_C leads to a reduction of mutual cooperation while the fraction of CD strategies slightly increase. The sensitivity to the population size is stronger for smaller populations and becomes weaker for larger population sizes.

The dependence on the mutation rate in strategies is investigated in Fig. (SI.9.e). The fraction of C and D strategies show small sensitivity to the mutation rate (upper part of Fig. (SI.9.e)). However, for very small mutation rates considered here ($\nu_\sigma = 0.001$) both the level of mutual cooperation (CC) and partial cooperation (CD) strongly increase by decreasing the mutation rate.

The dependence on the mutation rate in signals is investigated in Fig. (SI.9.f). Similarly to the model in which reproduction is done with a probability proportional to the payoff, the behavior seems to change depending on whether the mutation rate in signals is smaller or larger than the mutation rate in strategies. Both ρ_C and ρ_{CC} are maximized when ν_σ is comparable to ν_s. In other words, the level of cooperation is maximized when the time scale of evolution of signals and strategies are comparable and a gap in the time scale of the evolution of strategies and signals undermines cooperation. When $\nu_\sigma \ll \nu_s$, strategy densities become fast changing variables which adapt to the slowly changing signal distributions in the population. Such adaptation undermines cooperation, as defective strategies with high fitness are produced more often and grow in number by selection. In the opposite regime, when $\nu_s \ll \nu_\sigma$, agents have more chances to exploit cooperators by changing the probability with which they produce signals. In other words, strategies being a slowly changing variable with respect to the signal production probability, agents have the chance to exploit cooperators by changing their signal production probabilities in a way such that they get the most exploitation out of cooperators. This reduces cooperators' payoff the thus, decreases the frequency of cooperative strategies and mutual cooperation. To summarize, when there is a large gap between the time scale of change of strategies and signals, agents can use this gap, either to exploit the slowly changing profile of strategies by producing fit signals (when $\nu_s \ll \nu_\sigma$), or to switch to fitter strategies which defect more often against the prevalent signals (when $\nu_\sigma \ll \nu_s$). Both effects undermine cooperation, and the cooperation level is maximized when the time scale of change of signals and strategies are comparable.

Dependence on the strength of mutations in signals is investigated in Fig. (SI.9.g). Here. it seems that the fraction of strategies show very small sensitivity with respect to $d\sigma$.

SI. 5.2 The relation between signal fitness and signal density

In this section, we investigate the relation between signal fitness and signal density in the model in which agents reproduce with a probability proportional to the exponential of their payoff. In Fig. (SI.10), we plot the time average of signal density, as a function of time average signal payoff, for different games. In Fig.(SI.10.a) agents play a PD game, in Fig. (SI.10.b) the game is a TTD, in Fig. (SI.10.c) agent plays the SD game, in Fig. (SI.10.d), the game is BS, and finally in Fig. (SI.10.e) the game is the leader game. The time average density as a function of normalized apparent cost is plotted in the insets. Here, the time window over which an average is taken is equal to $t = 5000$ in all the cases. As can be seen, similarly to the model considered in the main text, when signal densities are plotted as a function of their apparent cost (insets) a confusing picture emerges. Not only signals with large cost are produced, signal densities seem to have no or little dependence on their apparent cost. While by plotting the signal densities as a function of their average payoff, in all the cases a strong increasing trend is observed. This results from the fact that signals are indirectly subject to selection based on their payoff. Consequently, signals with larger

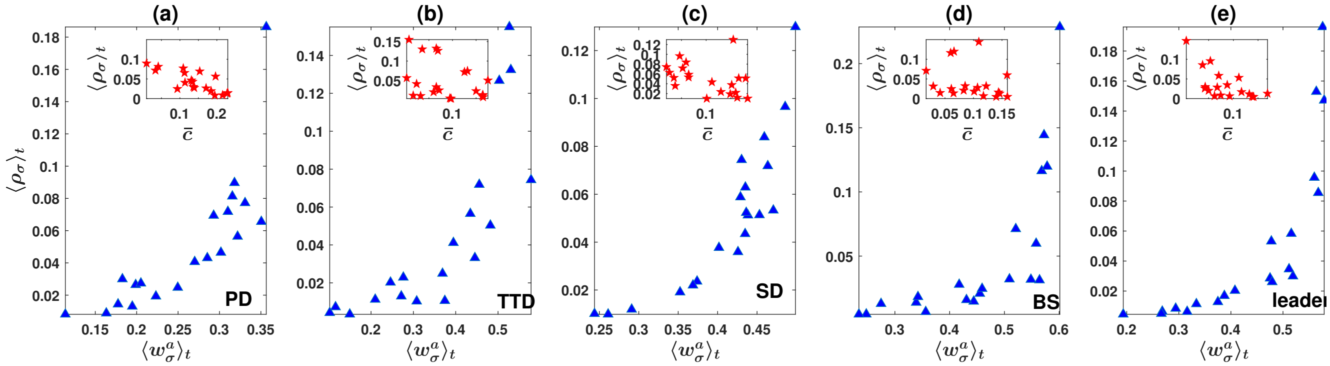

Figure SI.10. Increasing trend between signal density and signal payoff in the model in which individuals reproduce with a probability proportional to the exponential of their payoff. (a) to (e): Time averaged density of signals produced in the population $\langle \rho_\sigma \rangle_t$, as a function of their payoff averaged over the same time period for five different game structures. Insets show $\langle \rho_\sigma \rangle_t$, as a function of normalized cost (cost divided by mean payoff). When signal densities are plotted against apparent cost, a puzzling picture emerges: not only costly signals are produced, but also signals show little or no dependence on their cost. However, by plotting the signal density against their payoff, a strong pattern emerges: signal densities are distributed as an increasing function of their payoff. Here, in all the cases, time averages are taken over a window of length $t = 5000$.

payoff are selected more often and take a higher density.

In Table. (SI.4), we put the existence of a trend between signal densities and signal payoffs, and also between signal density and their apparent cost into a more quantitative test. For this purpose we appeal to two statistical tests to examine the presence of a trend: Spearman's rank correlation test and Mann-Kendal test [57,58]. The results of the tests are given in Table. (SI.4). Here, the Spearman's rank correlation coefficient between time average signal density and normalized apparent cost $r(\bar{c}, \langle \rho_\sigma \rangle_t)$, and also Spearman's rank correlation coefficient between $\langle \rho_\sigma \rangle_t$ and time average signal payoff $\langle w_\sigma^a \rangle_t$, denoted as $r(\langle w_\sigma^a \rangle_t, \langle \rho_\sigma \rangle_t)$, are given in the first rows. The p value of the Spearman's test p_r, and also the p value of the Mann-Kendall test p_{MK} are also given. Here an average over a time window of length $t = 5000$ is taken (the same quantities presented in Fig. (SI.10) are used for the tests). A p value smaller than 0.05 is conventionally considered as a success in establishing a trend and a p value larger than 0.05 is considered as a failure in establishing the trend. As can be seen, in the case of TTD, SD and BS, both tests fail to establish a trend between the signal density and signal cost, and in the case of PD and the leader game a weak trend is established. On the other hand, a very strong trend between signal density and signal payoff is established in all the cases. Furthermore, the trend between signal payoff and signal density is much stronger than the trend between signal density and their apparent cost.

The trend between signal density and signal payoff holds for other parameter values of the model in the case of all the games considered. As an example, here, we show the existence of this relation for different values of the model parameters in the case of the prisoner's dilemma game. For this purpose, for each of the model parameters, 3 different values are considered and r, p_r, and p_{MK} are calculate and presented in Table. (SI.5) (each first for the normalized cost and time average density, and then for time average fitness and time average density). As can be seen in most of the cases both tests fail to establish a trend between time average density and normalized apparent costs. While a strong or a very strong trend between time average density and time average fitness of signals is established using both tests. In addition, in almost all the cases the (increasing) trend between time average density and time average fitness is (much) stronger than the (decreasing) trend between time average density and normalized cost of signals. This means that signal fitness, explains signal densities in the population, much better than signal costs and can be seen as a solution to the puzzle posed by the presence of costly signals.

The only cases where, the tests suggest the trend between time average density and time average payoff, is similar (in magnitude) to the trend between time average density and normalized apparent cost, is the case of large cost ($c_{max} = 0.5$) and large β ($\beta = 50$). In the case of very high signal costs, such that the cost of signals is comparable or larger than the average payoff of the individuals, it does not pay for the individuals to produce very costly signals. Consequently, the cost differences between the signals can become more significant than the fitness differences resulting from the internal dynamics of the system and signal density show a similar trend (in magnitude) with respect to cost and payoff of signals. In the case of very large β, the fittest agents are selected with higher probability. It can happen that an individual contribute many offspring to the pool of the next generation. Under such condition the heterogeneity in the population and also the magnitude of the signal war is reduced. Consequently, agents who produce the cheapest signals obtain a higher payoff and are selected more often. This is why a rather strong trend between signal cost and signal density is observed in this case. This predicts that in a situation

n	PD	TTD	SD	BS	leader
$r(\bar{c}_\sigma, \langle\rho_\sigma\rangle_t)$	-0.48	-0.18	-0.43	-0.22	-0.49
$r(\langle w^a_\sigma\rangle_t, \langle\rho_\sigma\rangle_t)$	0.95	0.95	0.87	0.95	0.95
$p_r(\bar{c}_\sigma, \langle\rho_\sigma\rangle_t)$	2.8×10^{-2}	4.4×10^{-1}	5.6×10^{-2}	3.3×10^{-1}	2.5×10^{-2}
$p_r(\langle w^a_\sigma\rangle_t, \langle\rho_\sigma\rangle_t)$	1.1×10^{-10}	1.4×10^{-10}	4.4×10^{-7}	3.3×10^{-11}	6.2×10^{-11}
$p_{MK}(\bar{c}_\sigma, \langle\rho_\sigma\rangle_t)$	4.7×10^{-2}	4.9×10^{-1}	6.4×10^{-2}	3.1×10^{-1}	2.5×10^{-2}
$p_{MK}(\langle w^a_\sigma\rangle_t, \langle\rho_\sigma\rangle_t)$	1.7×10^{-7}	1.7×10^{-7}	2.1×10^{-5}	2.4×10^{-7}	4.9×10^{-7}

Table SI.4. Trend tests between signal density and signal payoff in the model in which individuals reproduce with a probability proportional to the exponential of their payoff, with different game structures. From top to down, Spearsman's rank correlation coefficient between apparent cost and time average density of signals $r(\bar{c}_\sigma, \langle\rho_\sigma\rangle_t)$, and between the time average fitness and time average density of signals $r(\langle w^a_\sigma\rangle_t, \langle\rho_\sigma\rangle_t)$, p value of the Spearman test between apparent cost and time average density of signals $p_r(\bar{c}_\sigma, \langle\rho_\sigma\rangle_t)$, and between time average fitness and time average density of signals $p_r(\langle w^a_\sigma\rangle_t, \langle\rho_\sigma\rangle_t)$, p value of Mann-Kendall test between apparent cost and time average density of signals $p_{MK}(\bar{c}_\sigma, \langle\rho_\sigma\rangle_t)$, and between time average fitness and time average density of signals $p_{MK}(\langle w^a_\sigma\rangle_t, \langle\rho_\sigma\rangle_t)$. Here, an average over a window of length $t = 5000$ is taken. In all the cases both tests strongly support a trend between fitness and density of signals, but in the case of TTD, and SD, fail to establish a trend between the apparent cost and density of signals. In all the cases, the trend between density and fitness is significantly stronger compared to the trend between apparent cost and density.

when small fitness differences can be determining in selection (such as the case where individuals produce offspring with a probability proportional to the exponential of their payoff, in the large β regime), costly signaling is more difficult to evolve.

In Table. (SI.5), the time window over which an average is taken is equal to $t = 5000$. The only exception is the case of $d\sigma = 0.01$, in which case $t = 10000$. Variation of the time window does not affect the results, as far as it is larger than a possible time lag between signal fitness and signal densities. This time lag results from the fact that when a signal's fitness increases, it takes some time until enough mutations happen and grow for the density of that signal to increase as well. Just as in the model in which selection occurs with a probability proportional to payoff, in most of the parameter values considered here, this time lag is rather small (less than 100 time steps). However, for small $d\sigma$ this time lag can be larger, as it takes longer time for mutations to be accumulated enough for the signal densities to follow signal fitness.

v_s	0.001	0.05	1	v_σ	0.001	0.05	1
$r(\bar{c}_\sigma, \langle\rho_\sigma\rangle_t)$	-0.29	-0.12	-0.65		-0.50	-0.24	-0.21
$r(\langle w^a_\sigma\rangle_t, \langle\rho_\sigma\rangle_t)$	0.82	0.91	0.78		0.76	0.87	0.92
$p_r(\bar{c}_\sigma, \langle\rho_\sigma\rangle_t)$	2.1×10^{-1}	6.0×10^{-1}	1.9×10^{-3}		2.2×10^{-2}	2.9×10^{-1}	3.5×10^{-1}
$p_r(\langle w^a_\sigma\rangle_t, \langle\rho_\sigma\rangle_t)$	7.3×10^{-6}	1.7×10^{-8}	3.4×10^{-5}		8.8×10^{-5}	3.2×10^{-7}	4.6×10^{-9}
$p_{MK}(\bar{c}_\sigma, \langle\rho_\sigma\rangle_t)$	2.3×10^{-1}	5.8×10^{-1}	4.8×10^{-3}		1.7×10^{-3}	2.8×10^{-1}	2.5×10^{-1}
$p_{MK}(\langle w^a_\sigma\rangle_t, \langle\rho_\sigma\rangle_t)$	3.7×10^{-5}	3.4×10^{-6}	3.4×10^{-5}		2.4×10^{-4}	1.5×10^{-5}	4.9×10^{-7}

dc	0.05	0.25	0.5	ds	0.01	0.1	0.5
$r(\bar{c}_\sigma, \langle\rho_\sigma\rangle_t)$	-0.05	-0.64	-0.59		-0.53	-0.71	-0.44
$r(\langle w^a_\sigma\rangle_t, \langle\rho_\sigma\rangle_t)$	0.92	0.93	0.60		0.80	0.93	0.90
$p_r(\bar{c}_\sigma, \langle\rho_\sigma\rangle_t)$	8.1×10^{-1}	2.1×10^{-3}	4.6×10^{-3}		1.6×10^{-2}	4.0×10^{-4}	4.9×10^{-2}
$p_r(\langle w^a_\sigma\rangle_t, \langle\rho_\sigma\rangle_t)$	3.8×10^{-9}	1.2×10^{-9}	5.8×10^{-2}		1.9×10^{-5}	2.2×10^{-9}	2.7×10^{-8}
$p_{MK}(\bar{c}_\sigma, \langle\rho_\sigma\rangle_t)$	7.2×10^{-1}	2.1×10^{-3}	1.2×10^{-2}		1.5×10^{-2}	8.3×10^{-4}	7.4×10^{-2}
$p_{MK}(\langle w^a_\sigma\rangle_t, \langle\rho_\sigma\rangle_t)$	1.8×10^{-6}	4.9×10^{-7}	2.8×10^{-5}		8.6×10^{-5}	3.5^{-7}	2.5×10^{-6}

n	5	20	50	N	100	400	1600
$r(\bar{c}_\sigma, \langle\rho_\sigma\rangle_t)$	-0.6	-0.48	-0.42		-0.36	-0.48	-0.29
$r(\langle w^a_\sigma\rangle_t, \langle\rho_\sigma\rangle_t)$	1	0.95	0.92		0.96	0.95	0.77
$p_r(\bar{c}_\sigma, \langle\rho_\sigma\rangle_t)$	2.8×10^{-1}	2.8×10^{-2}	1.9×10^{-3}		1.1×10^{-1}	2.8×10^{-2}	2.0×10^{-1}
$p_r(\langle w^a_\sigma\rangle_t, \langle\rho_\sigma\rangle_t)$	0	1.1×10^{-10}	0		1.7×10^{-12}	1.1×10^{-10}	6.4×10^{-5}
$p_{MK}(\bar{c}_\sigma, \langle\rho_\sigma\rangle_t)$	4.6×10^{-1}	4.7×10^{-2}	2.1×10^{-3}		1.6×10^{-1}	4.7×10^{-2}	2.5×10^{-1}
$p_{MK}(\langle w^a_\sigma\rangle_t, \langle\rho_\sigma\rangle_t)$	2.7×10^{-2}	1.7×10^{-7}	3.7×10^{-15}		8.6×10^{-8}	1.7×10^{-7}	8.6×10^{-5}

β	0.1	1	50
$r(\bar{c}_\sigma, \langle\rho_\sigma\rangle_t)$	-0.34	-0.49	-0.72
$r(\langle w^a_\sigma\rangle_t, \langle\rho_\sigma\rangle_t)$	0.45	0.94	0.61
$p_r(\bar{c}_\sigma, \langle\rho_\sigma\rangle_t)$	1.3×10^{-1}	2.5×10^{-2}	3.1×10^{-4}
$p_r(\langle w^a_\sigma\rangle_t, \langle\rho_\sigma\rangle_t)$	4.2×10^{-2}	2.4×10^{-10}	4.6×10^{-3}
$p_{MK}(\bar{c}_\sigma, \langle\rho_\sigma\rangle_t)$	1.6×10^{-1}	2.5×10^{-2}	2.8×10^{-4}
$p_{MK}(\langle w^a_\sigma\rangle_t, \langle\rho_\sigma\rangle_t)$	4.7×10^{-2}	3.5×10^{-7}	3.9×10^{-3}

Table SI.5. Dependence of the trend between signal density and signal payoff on model parameters in the model in which individuals reproduce with a probability proportional to the exponential of their payoff. From top to down, trend test between time average density of signals $\langle\rho_\sigma\rangle_t$, and normalized apparent cost $\bar{c}$, and between $\langle\rho_\sigma\rangle_t$ and time average payoff of signal $\langle w^a_\sigma\rangle_t$ for different parameter values in the prisoner's dilemma game. From top to down, Spearman's rank correlation coefficient between $\langle\rho_\sigma\rangle_t$ and $\bar{c}$, and between $\langle\rho_\sigma\rangle_t$ and $\langle w^a_\sigma\rangle_t$, p value of the Spearman test between $\langle\rho_\sigma\rangle_t$ and $\bar{c}$, and between $\langle\rho_\sigma\rangle_t$ and $\langle w^a_\sigma\rangle_t$,, and finally, p value of the Mann-Kendall test between $\langle\rho_\sigma\rangle_t$ and $\bar{c}$, and between $\langle\rho_\sigma\rangle_t$ and $\langle w^a_\sigma\rangle_t$. In all the cases both tests strongly support a trend between fitness and density of signals, but in most of the cases, fail to establish a trend between the apparent cost and density of signals. In all the cases, the trend between density and realized fitness is significantly stronger compared to the trend between apparent cost and density.
